# Guselkumab binding to CD64^+^ IL-23–producing myeloid cells enhances potency for neutralizing IL-23 signaling

**DOI:** 10.3389/fimmu.2025.1532852

**Published:** 2025-03-12

**Authors:** Kacey L. Sachen, Deepa Hammaker, Indra Sarabia, Brian Stoveken, John Hartman, Kristin L. Leppard, Nicholas A. Manieri, Phuc Bao, Carrie Greving, Eilyn R. Lacy, Matthew DuPrie, Joshua Wertheimer, Janise D. Deming, Joseph Brown, Amy Hart, He (Hurley) Li, Tom C. Freeman, Brice Keyes, Kristen Kohler, Ian White, Nathan Karpowich, Ruth Steele, M. Merle Elloso, Steven Fakharzadeh, Kavitha Goyal, Frédéric Lavie, Maria T. Abreu, Matthieu Allez, Raja Atreya, Robert Bissonnette, Kilian Eyerich, James G. Krueger, Dennis McGonagle, Iain B. McInnes, Christopher Ritchlin, Anne M. Fourie

**Affiliations:** ^1^ Johnson & Johnson, San Diego, CA, United States; ^2^ Johnson & Johnson, Spring House, PA, United States; ^3^ Johnson & Johnson, Horsham, PA, United States; ^4^ Johnson & Johnson, Paris, France; ^5^ University of Miami, Leonard M. Miller School of Medicine, Miami, FL, United States; ^6^ Hôpital Saint-Louis, Université Paris Cité, Paris, France; ^7^ Department of Medicine 1, Erlangen University Hospital, Friedrich-Alexander-Universität Erlangen-Nürnberg, Erlangen, Germany; ^8^ Innovaderm Research Inc, Montréal, QC, Canada; ^9^ Medical Center, University of Freiburg, Freiburg, Germany; ^10^ Department of Medicine – Division of Dermatology and Venereology, Karolinska Institute, Stockholm, Sweden; ^11^ Laboratory for Investigative Dermatology, The Rockefeller University, New York, NY, United States; ^12^ Leeds Biomedical Research Centre, University of Leeds, Leeds, United Kingdom; ^13^ College of Medical, Veterinary, and Life Sciences, University of Glasgow, Glasgow, United Kingdom; ^14^ Center for Musculoskeletal Research, Allergy, Immunology, and Rheumatology Division, University of Rochester, Rochester, NY, United States

**Keywords:** IL-23, IL-23p19 subunit inhibitors, guselkumab, risankizumab, CD64, *in vitro* cellular assays, RNA sequencing, immune-mediated inflammatory diseases

## Abstract

IL-23 is implicated in the pathogenesis of immune-mediated inflammatory diseases, and myeloid cells that express Fc gamma receptor 1 (FcγRI or CD64) on their surface have been recently identified as a primary source of IL-23 in inflamed tissue. Our complementary analyses of transcriptomic datasets from psoriasis and IBD showed increased expression of CD64 and IL-23 transcripts in inflamed tissue, and greater abundance of cell types with co-expression of CD64 and IL-23. These findings led us to explore potential implications of CD64 binding on the function of IL-23–targeting monoclonal antibodies (mAbs). Guselkumab and risankizumab are mAbs that target the IL-23p19 subunit. Guselkumab has a native Fc domain while risankizumab contains mutations that diminish binding to FcγRs. In flow cytometry assays, guselkumab, but not risankizumab, showed Fc-mediated binding to CD64 on IFNγ-primed monocytes. Guselkumab bound CD64 on IL-23–producing inflammatory monocytes and simultaneously captured IL-23 secreted from these cells. Guselkumab binding to CD64 did not induce cytokine production. In live-cell confocal imaging of CD64^+^ macrophages, guselkumab, but not risankizumab, mediated IL-23 internalization to low-pH intracellular compartments. Guselkumab and risankizumab demonstrated similar potency for inhibition of IL-23 signaling in cellular assays with exogenous addition of IL-23. However, in a co-culture of IL-23–producing CD64^+^ THP-1 cells with an IL-23–responsive reporter cell line, guselkumab demonstrated Fc-dependent enhanced potency compared to risankizumab for inhibiting IL-23 signaling. These *in vitro* data highlight the potential for guselkumab binding to CD64 in inflamed tissue to contribute to the potent neutralization of IL-23 at its cellular source.

## 1 Introduction

IL-23 is a pro-inflammatory member of the IL-12 cytokine family and plays a pivotal role in the pathogenesis of immune-mediated inflammatory diseases, including psoriatic disease (psoriasis [PsO] and psoriatic arthritis [PsA]) and inflammatory bowel disease (IBD; Crohn’s disease [CD] and ulcerative colitis [UC]) (1–5). IL-23 is a heterodimeric cytokine composed of a p19 subunit specific to IL-23 (IL-23p19) and a p40 subunit (IL-23p40) shared with IL-12 (6). Myeloid cells, including monocytes, macrophages, dendritic cells, and epidermal Langerhans cells, are the major sources of IL-23, the production of which is primarily induced through toll-like receptor (TLR) activation (6–10).

IL-23 signals through a heterodimeric cell surface receptor consisting of IL-23R and IL-12Rβ1 (11). While multiple hypotheses are proposed regarding the precise mechanism of IL-23 binding to its cognate receptor subunits, it is generally accepted that the IL-23p19 subunit binds to IL-23R and the IL-23p40 subunit binds to IL-12Rβ1 (12–15). IL-23 binding to its receptor activates JAK and STAT signaling molecules, with STAT3 phosphorylation as a key proximal signaling event (11). IL-23 receptors are expressed on both innate and adaptive immune cells, including innate lymphoid cells, subsets of memory T cells, natural killer T cells, γδ T cells, and mucosal-associated invariant T cells (16). The production of IL-23 by tissue-resident myeloid cells promotes survival and expansion of T helper type 17 cells and activates IL-23 receptor-expressing cells to produce downstream effector cytokines (e.g., IL-17A, IL-17F, and IL-22), contributing to local tissue inflammation (17). Additionally, IL-23 is important for survival of tissue-resident memory T cells and suppresses regulatory T cell differentiation, both of which have been linked to the chronicity of immune-mediated inflammatory diseases (18–20).

The efficacy of selective blockade of IL-23 with therapeutic monoclonal antibodies (mAbs) targeting the IL-23p19 subunit, such as guselkumab and risankizumab, has been well-established in PsO (21, 22), PsA (21, 22), CD (22–24), and UC (21, 22). Guselkumab is a fully human IgG1, λ antibody containing a wild-type Fc domain (25), whereas risankizumab is a humanized IgG1, κ antibody with L234A, L235A (LALA) mutations in the Fc domain that diminish its ability to interact with Fcγ receptors (FcγRs) (26).

FcγRs are a class of cell surface receptors that bind to the Fc portion of IgG and can mediate either activating or inhibitory signals when cross-linked by polyvalent immune complexes. The majority of FcγRs are classified as low-affinity and can only bind to IgG in multimeric antibody-antigen immune complexes. FcγRI, also known as CD64, is the only FcγR that binds with high affinity to monomeric IgG molecules, including IgG1, IgG3, and IgG4 (27, 28). As IL-23 is a secreted cytokine and mAb binding to IL-23 would not form polyvalent immune complexes, FcγR binding has not been thought to contribute to the therapeutic mechanism of action of mAbs targeting IL-23. However, recent developments identifying CD64^+^ myeloid cells as a primary source of IL-23 in inflamed tissue prompted us to consider how mAb binding to CD64 could impact neutralization of IL-23 (20, 29, 30).

Flow cytometric analyses of CD45^+^ cells isolated from lesional skin of patients with PsO revealed a significant increase in mononuclear phagocytes expressing CD64 relative to non-lesional skin; these CD64^+^ myeloid cells accounted for 80% of the IL-23p19^+^ cells in lesional skin (20). While the specific cellular source of IL-23 in PsA synovial tissue has yet to be defined, it has been demonstrated that CD64^+^ myeloid cells residing in uninflamed enthesis can produce IL-23 following stimulation with TLR ligands (31, 32). Patients with PsA also have an increase in the percentage of peripheral blood monocytes with CD64 expression, which correlates with joint disease activity (33). In IBD, flow cytometric analyses of myeloid cells from both CD and UC colon tissue identified CD64^+^ myeloid cells as a primary source of IL-23 in inflamed tissue (29, 30). Similarly, in the *Helicobacter hepaticus* infection model of IBD, expression of IL-23 was primarily restricted to CD64^+^ myeloid cells, and specific knockout of IL-23p19 expression in CD11c cells, which also express CD64, led to a striking reduction in the severity of colitis (34, 35). Thus, across disease states, CD64^+^ myeloid cells have been identified as essential sources of IL-23 driving disease pathology.

In this study, the relationship of CD64 expression at the transcriptional level to tissue inflammation and IL-23 expression was further explored in bulk and single-cell analyses. Furthermore, guselkumab and risankizumab, which both bind and neutralize IL-23 but have differences in their Fc domains that affect binding to FcγRs, were compared in biophysical and *in vitro* cellular assays to explore how mAb functions are shaped by their unique molecular attributes. We found that guselkumab binding to CD64 on IL-23–producing myeloid cells via the Fc domain enabled capture of IL-23 at the cellular source of production, leading to internalization and trafficking of IL-23 to endolysosomal compartments, and enhanced potency for neutralization of IL-23 signaling.

## 2 Results

### 2.1 CD64 and IL-23 transcripts are elevated in inflamed tissues and are co-expressed by myeloid cells in PsO and IBD

In order to complement previous observations of CD64 and IL-23 protein co-expression (20, 29, 30), we analyzed gene expression in bulk RNA-sequencing datasets from patients with PsO, CD, and UC (36, 37) to investigate expression of RNA encoding CD64 (*FCGR1A*), IL-23p19 (*IL23A*), and IL-23p40 (*IL12B*) in inflamed and uninflamed tissue. We found significantly increased expression of *FCGR1A, IL23A*, and *IL12B* in lesional skin from patients with PsO, compared to non-lesional skin. The lower expression of *FCGR1A, IL23A*, and *IL12B* observed in non-lesional skin samples from patients with PsO was comparable to that in skin from healthy subjects (**Supplementary Figure S1A**). Similar results were observed in our analyses of rectal biopsy datasets from patients with CD and UC. Expression of *FCGR1A*, *IL23A*, and *IL12B* were all significantly increased in inflamed IBD samples, compared to both non-inflamed IBD and non-IBD control samples (**Supplementary Figure S1B**).

**Figure 1:**
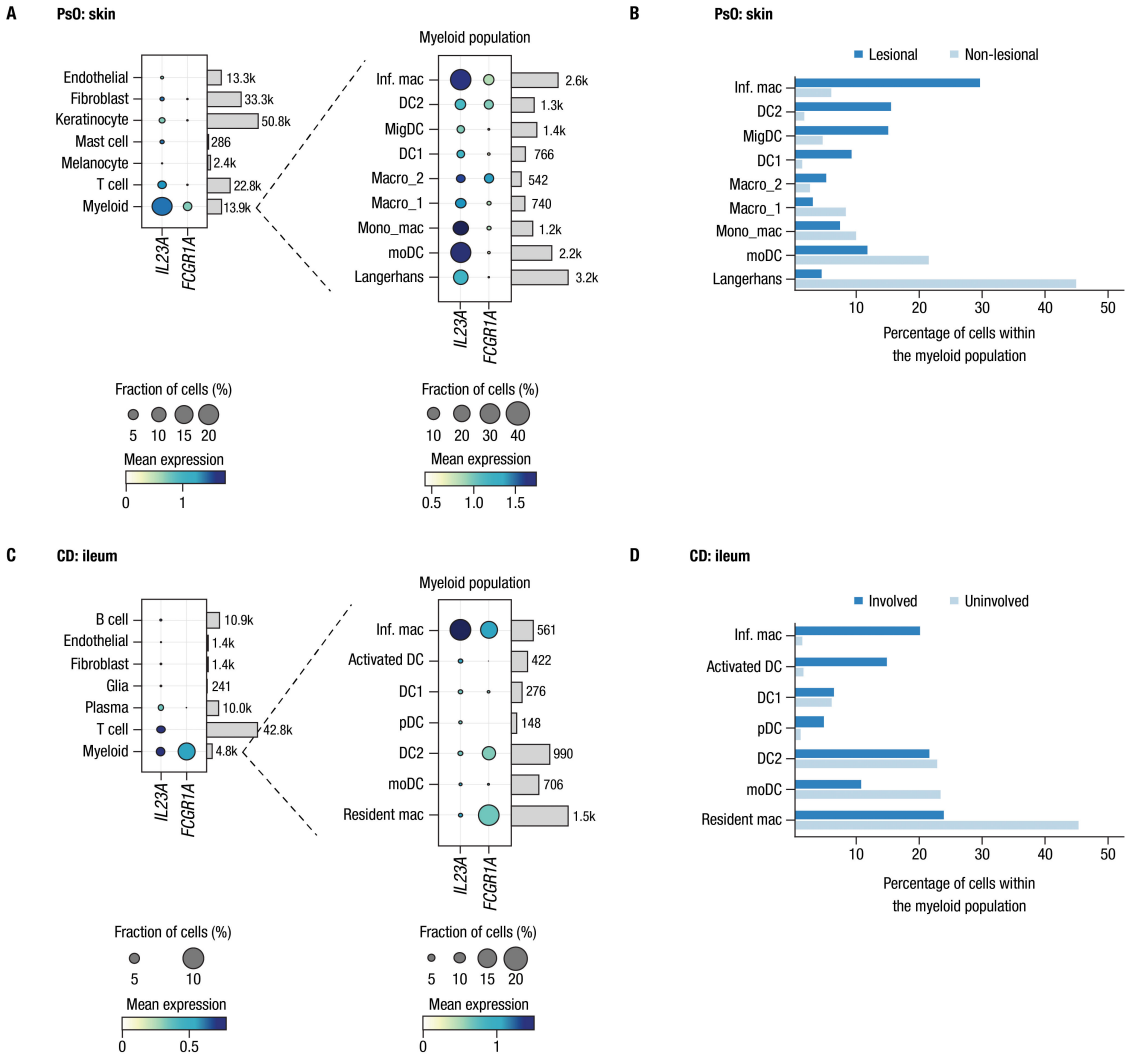
CD64 and IL-23 transcripts are elevated in inflamed tissues and are co-expressed by myeloid cells in PsO and IBD. Assessments of *FCGR1A* and *IL23A* expression from single-cell RNA-sequencing datasets from biopsies of patients with PsO and CD. Panel **(A)** PsO skin (n = 3 patients); panel **(C)** ileal tissue from patients with CD (n = 9 patients). Dot plots indicate mean expression level (denoted by the color of dot) and the fraction of cells expressing the *IL23A* and *FCGR1A* transcripts across various cell types (denoted by the size of dot). Cell types are provided to the left of dot plots; number of cells for each type in data used in these analyses are shown on the right. Expanded views of gene expression within the myeloid cell population are presented to the right of each plot. Bars attached to each dot plot indicate the count of cells from each identified cell population. Panels **(B, D)** show the percentage of cells in the myeloid population from lesional/involved and non-lesional/uninvolved tissues in PsO skin and CD ileum, respectively. Inf. mac, inflammatory macrophage; DC, dendritic cell; MigDC, migratory dendritic cell; Macro, macrophage; Mono_mac, monocyte-derived macrophage; moDC, monocyte-derived dendritic cell; pDC, plasmacytoid dendritic cell.

We further investigated co-expression of *IL23A* and *FCGR1A* transcripts in tissues relevant to PsO and CD by analyzing single-cell transcriptomic data available from previous publications that investigated inflammatory skin diseases and CD, leveraging the annotations for cells and tissue types described in the original publications (38, 39). Expression of *IL12B* transcript was too low to enable robust analysis. In both skin of patients with PsO and ileum samples from patients with CD, *FCGR1A* was predominantly expressed in myeloid cells, which also expressed *IL23A* (**Figures 1A, C**). Among myeloid cells, the primary subtype expressing both *IL23A* and *FCGR1A* in both PsO skin and CD ileum was inflammatory macrophages. Moreover, in PsO skin, co-expression was also observed in a subset of macrophages (Macro_2) with gene set enrichment related to the regulation of angiogenesis, leukocyte chemotaxis, and TGFβ signaling, and a dendritic cell subset (DC2) associated with Th2 and Th17 responses (38, 40). Notably, these 3 cell types were more prevalent in lesional compared to non-lesional skin from patients with PsO (**Figure 1B**). Inflammatory macrophages expressing both *IL23A* and *FCGR1A* were more prevalent in involved ileal tissue compared to uninvolved tissues from patients with CD (**Figure 1D**).

Having established the connection at the transcriptional level between CD64-expressing myeloid cells, inflammation, and IL-23 production, we explored the potential implications of CD64 binding on the function of IL-23–targeting therapeutic mAbs. To this end, we focused on guselkumab and risankizumab, both of which target the IL-23p19 subunit and are approved, or under investigation, for the treatment of PsO, PsA, CD, and UC. We characterized and compared key functional attributes of the antigen-binding and Fc regions of these antibodies in a series of *in vitro* studies.

### 2.2 Guselkumab binds to CD64 in an Fc-dependent manner and can simultaneously capture IL-23

We evaluated binding of guselkumab and risankizumab to specific FcγRs using a competitive binding assay in which individual recombinant FcγRs were expressed in HEK293 cells. Across the panel of FcγRs assessed, guselkumab demonstrated the strongest binding to CD64 (**Supplementary Figure S2A**). A similar pattern of binding was observed for the hIgG1 IC (**Supplementary Figure S2C**). These observations were consistent with the classification of CD64 as the only FcγR demonstrating high-affinity binding to monomeric IgG1 (27). In contrast, risankizumab, which contains LALA mutations in the Fc domain that diminish binding to FcγRs (26), showed negligible binding to any FcγR (**Supplementary Figure S2B**).

**Figure 2:**
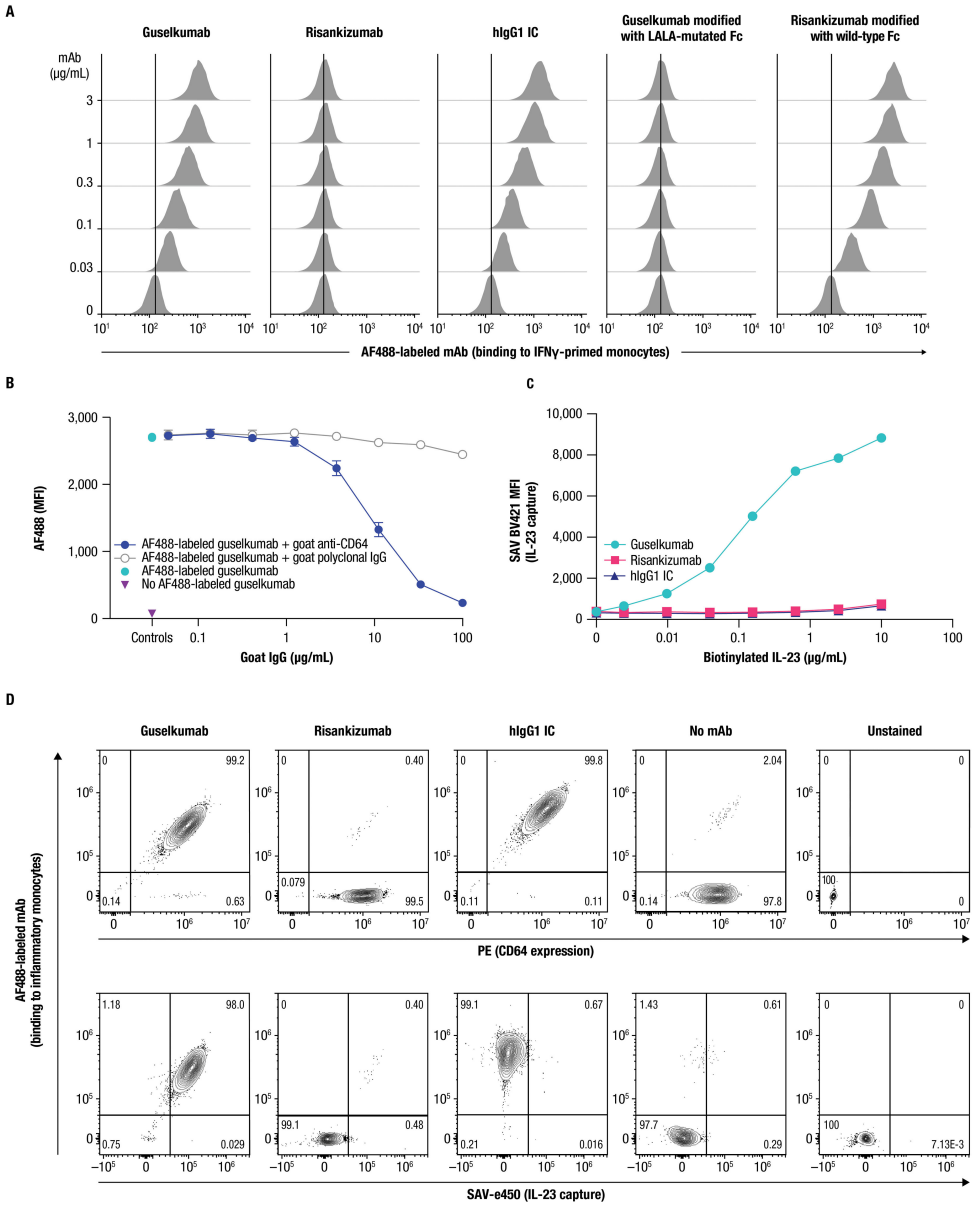
Guselkumab binds to CD64 in an Fc-dependent manner and can simultaneously capture IL-23. **(A)** IFNγ-primed monocytes were incubated with a dose titration of AF488-labeled guselkumab, risankizumab, hIgG1 IC, guselkumab modified to contain LALA mutations in the Fc domain, or risankizumab modified to contain a wild-type Fc domain. mAb binding to cells was assessed by flow cytometry. **(B)** IFNγ-primed monocytes were pre-incubated with a dose titration of goat polyclonal antibodies specific to CD64 or goat polyclonal IgG control. Cells were then incubated with 1µg/mL AF488-labeled guselkumab and binding of guselkumab was assessed by flow cytometry. **(C)** IFNγ-primed monocytes were incubated with 0.1 µg/mL guselkumab, risankizumab, or hIgG1 IC. Unbound mAb was washed away and cells were incubated with a dose-titration of biotinylated recombinant IL-23. Captured IL-23 was detected with BV421-labeled streptavidin, and cells were analyzed by flow cytometry. **(D)** Primary human monocytes were differentiated into inflammatory monocytes by culturing in the presence of GM-CSF and IFNγ for 6 days. Cells were then cultured in the presence of 0.3 µg/mL of AF488-labeled guselkumab, risankizumab, or hIgG1 IC and stimulated with TLR ligands LPS and R848 to promote endogenous secretion of IL-23. Following a 20-hour incubation, cells were washed and captured IL-23 was detected with a biotinylated antibody specific to IL-23p40 and SAVe450. Cells were analyzed by flow cytometry and data plotted to show correlations between CD64 expression, mAb binding, and capture of IL-23. Rare outlier events are due to cross-sample carryover during sample acquisition. All data shown are representative of ≥3 independent experiments.

Next, we examined binding of guselkumab to endogenous CD64 expressed by primary myeloid cells. Primary human monocytes display constitutive, low-level expression of CD64, which can be upregulated following an inflammatory stimulus, such as exposure to IFNγ (41, 42). Using flow cytometry, we confirmed upregulation of CD64 on the surface of IFNγ-primed primary human monocytes (**Supplementary Figure S3A**), which were then used to assess binding of mAbs labeled with Alexa Fluor 488 (AF488). Guselkumab and human IgG1 isotype control (hIgG1 IC) showed dose-dependent binding to IFNγ-primed monocytes, while risankizumab did not exhibit binding at any concentration evaluated (**Figure 2A**). To confirm that the differential capacity of guselkumab and risankizumab to bind IFNγ-primed monocytes was attributable to their distinct Fc domains, we engineered a variant of guselkumab containing LALA mutations in the Fc domain and a variant of risankizumab with a wild-type Fc domain. The LALA mutations abrogated the ability of guselkumab to bind to IFNγ-primed monocytes, while the wild-type Fc domain enabled risankizumab binding.

The binding of guselkumab to IFNγ-primed primary human monocytes positively correlated with the level of CD64 (**Supplementary Figure S4**). A correlation was less apparent for CD32 expression and, while CD16-expressing cells were infrequent in this cell population, the level of CD16 expression did not appear to influence guselkumab binding. Furthermore, a goat polyclonal antibody specific to CD64 blocked binding of guselkumab in a dose-dependent manner (**Figure 2B**). Taken together, these data suggest that binding of guselkumab to IFNγ-primed monocytes primarily occurred through interaction between the Fc domain and CD64.

We next evaluated the ability of CD64-anchored guselkumab to simultaneously capture IL-23 on IFNγ-primed monocytes using flow cytometry. In this assay, the IL-23 heterodimeric protein contained a biotin-tagged p19 subunit, and capture of IL-23 was detected with fluorescently labeled streptavidin. Cells incubated with guselkumab displayed dose-dependent capture of exogenous IL-23 added to the cell suspension (**Figure 2C**). This was not observed for cells that had been incubated with risankizumab or hIgG1 IC. These data demonstrated the ability of guselkumab to bind IL-23 while being anchored to CD64.

We further explored whether guselkumab bound to cell surface CD64 could simultaneously capture endogenous IL-23 produced locally by the same cells. CD14^+^ monocytes were cultured in the presence of GM-CSF and IFNγ for 6 days to induce differentiation into CD64^+^ inflammatory monocyte-like cells that produce IL-23 in response to TLR stimulation (43). In our assay, differentiated CD64^+^ inflammatory monocytes were stimulated with lipopolysaccharide (LPS) and resiquimod (R848) to promote production of IL-23 in the presence of AF488-labeled guselkumab, risankizumab, or hIgG1 IC. After 20 hours, cells were washed, and bound AF488-labeled antibody, CD64 expression, and captured IL-23 were measured by flow cytometry. Captured IL-23 was detected with a biotinylated antibody specific to the IL-23p40 subunit and fluorescent streptavidin. Consistent with results described above, the extent of guselkumab and hIgG1 IC binding correlated with the level of CD64 expression, while cell surface binding of risankizumab was not detected (**Figure 2D** top panel and **Supplementary Figure S5**). Furthermore, cells cultured in the presence of guselkumab demonstrated capture of endogenous, locally produced IL-23, the extent of which correlated with the level of cell surface–bound guselkumab (**Figure 2D** bottom panel and **Supplementary Figure S5**). As expected, cells cultured in the presence of risankizumab or hIgG1 IC did not demonstrate capture of IL-23. Thus, these data confirm the ability of guselkumab to not only bind to inflammatory monocyte-like cells via CD64, but also simultaneously capture IL-23 produced by these cells.

### 2.3 Guselkumab binding to CD64 does not induce cytokine secretion by CD64^+^ myeloid cells

**Figure 3:**
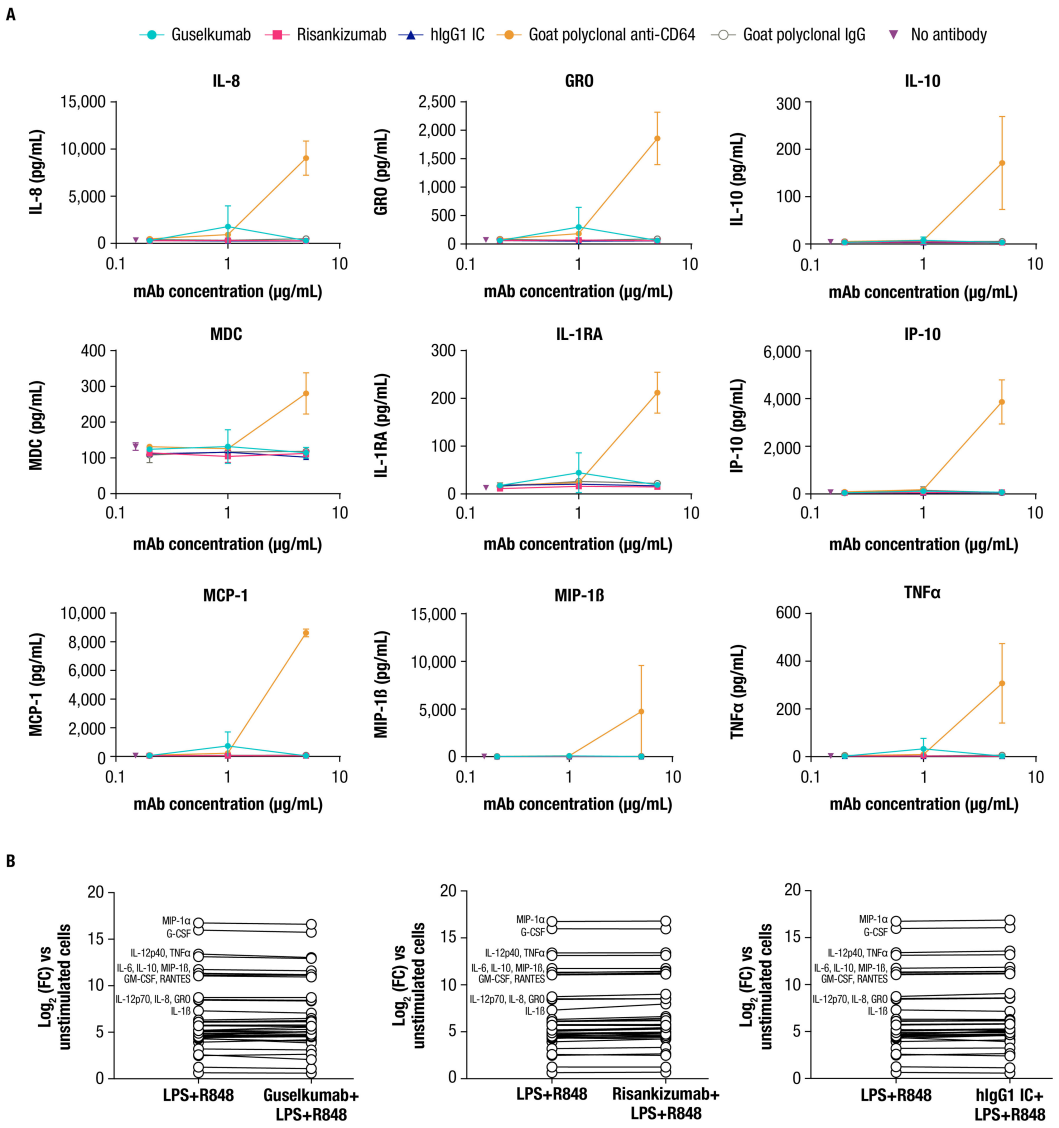
Guselkumab binding to CD64 does not induce or enhance cytokine secretion by CD64^+^ myeloid cells. **(A)** IFNγ-primed monocytes were cultured for 24 hours in the presence of guselkumab, risankizumab, hIgG1 IC, goat polyclonal antibodies specific to CD64, or goat polyclonal IgG control. A Milliplex 41-plex human cytokine bead assay was used to quantitate secreted cytokines in culture supernatants. Only cytokines with detectable secretion are shown. Data shown are representative of 3 independent experiments. **(B)** Inflammatory monocytes were stimulated with LPS and R848 and incubated with 0.3 µg/mL unlabeled mAb for 20 hours. A Milliplex 41-plex human cytokine bead assay was used to quantitate secreted cytokines in culture supernatants. Data are plotted at log_2_ fold change versus unstimulated cells. Cytokines with greatest induction are annotated in the graph. Data shown are representative of 2 independent experiments.

CD64 is classified as an activating FcγR and has been reported to mediate effector functions following cross-linking upon binding to polyvalent antibody-antigen immune complexes (44). While guselkumab binding is not expected to mediate cross-linking of CD64, we explored the potential for guselkumab binding to CD64 on monocytes to induce cellular activation by measuring cytokine production. IFNγ-primed monocytes were cultured in the presence of guselkumab, hIgG1 IC, or risankizumab for 24 hours, and culture supernatants were evaluated for secreted cytokines with a 41-plex human cytokine bead assay. In the presence of goat polyclonal antibody specific to CD64, which would be expected to cross-link receptors, induction of cytokine secretion was observed (i.e., IL-8, GRO, IL-10, MDC, IL-1RA, IP-10, MCP-1, MIP-1β, and TNFα), indicating cellular activation (**Figure 3A**). The presence of guselkumab or hIgG1 IC, both of which bind to CD64, did not induce cytokine production by IFNγ-primed monocytes, similar to the outcome for risankizumab, which does not bind to CD64.

We also determined whether guselkumab binding to CD64 affected cytokine secretion by the LPS- and R848-stimulated inflammatory monocytes used to demonstrate guselkumab binding to CD64 and simultaneous capture of locally produced IL-23 (**Figure 2D**). Cells were cultured as previously described with unlabeled antibodies, and culture supernatants were evaluated for secreted cytokines. LPS and R848 stimulation led to robust induction of numerous cytokines compared with unstimulated cells (**Figure 3B**). The presence of guselkumab did not alter the LPS- and R848-induced cytokine secretion, similar to risankizumab or hIgG1 IC, which did not bind to CD64 or IL-23, respectively.

### 2.4 Guselkumab bound to CD64 on inflammatory monocytes via its Fc domain can mediate internalization of IL-23 to endolysosomal compartments

CD64 has been reported to internalize when bound to monomeric hIgG1, or when aggregated with antibody-antigen immune complexes or cross-linking antibodies (45–49). To determine the fate of CD64-bound guselkumab–IL-23 complexes, we utilized live-cell confocal microscopy. Primary human monocytes were differentiated into classically activated, pro-inflammatory, CD64-expressing macrophages by culturing in the presence of GM-CSF for 6 days followed by IFNγ for 24 hours (50) (**Supplementary Figure S3B**). Cells were then incubated with pHrodo Red–labeled IL-23 alone, or with AF488-labeled guselkumab, risankizumab, or hIgG1 IC, and imaged over 24 hours. The pHrodo Red label on IL-23 does not fluoresce at neutral pH but becomes fluorescent under acidic conditions, such as within intracellular endolysosomal compartments.

**Figure 4:**
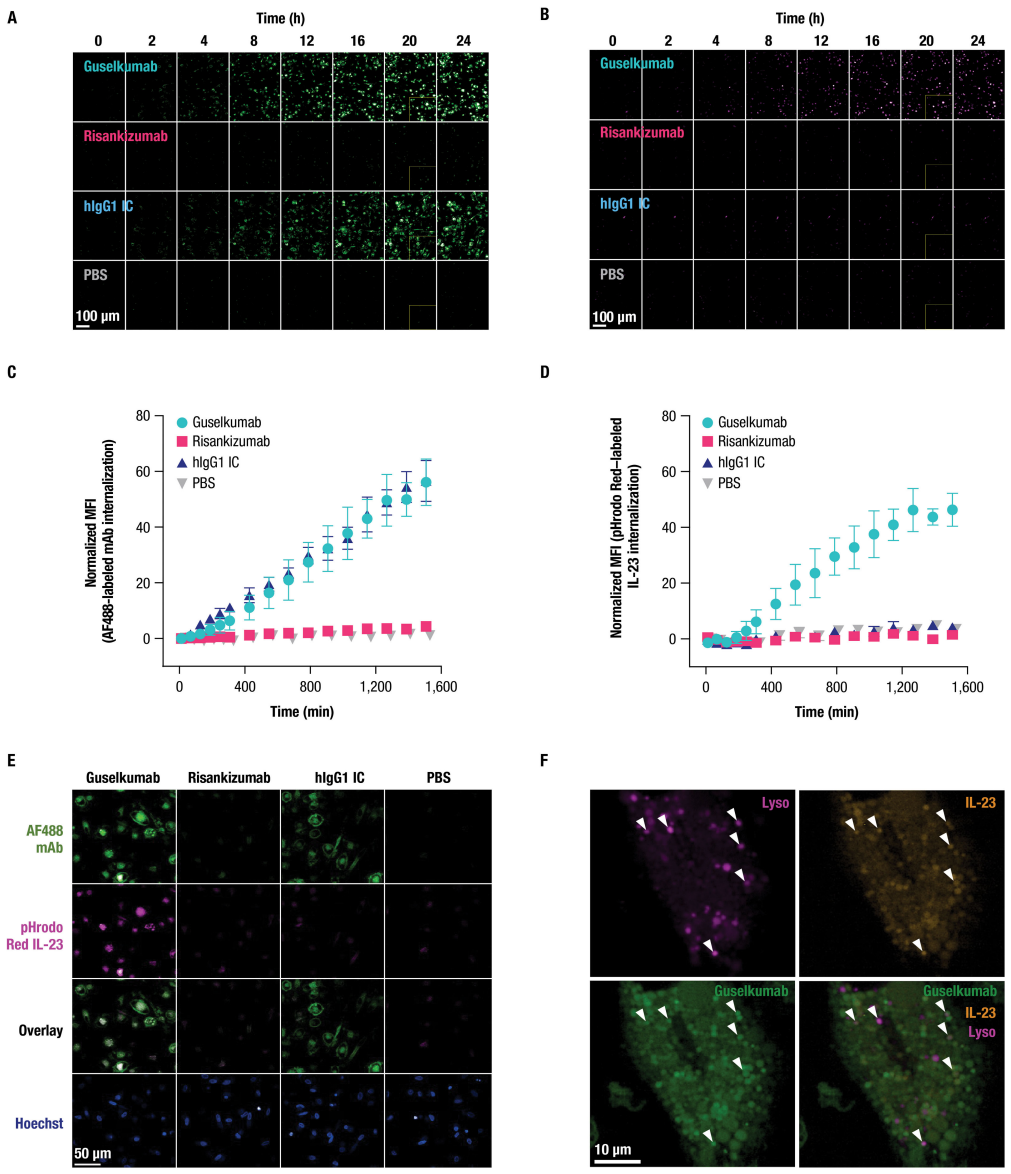
Guselkumab bound to CD64 on inflammatory monocytes via its Fc domain can mediate internalization of IL-23 to endolysosomal compartments. **(A–F)** Primary human monocytes were differentiated into CD64-expressing macrophages by culturing in the presence of GM-CSF for 6 days and were then primed overnight with IFNγ. Live cell fluorescence imaging was performed with high-throughput spinning disk confocal microscopy. **(A–E)** Macrophages were incubated with 10 nM IL-23 labeled with a pH-sensitive fluor, pHrodo Red, and 10 nM AF488-labeled mAb. Staining with CellTracker Deep Red and Hoechst33342 were used to define cytoplasmic and nuclear regions, respectively. **(A)** Time-lapse imaging shows time-dependent binding and internalization of AF488-labeled mAbs into macrophages (shown in green). Scale bar is 100 µm. **(B)** Time-lapse imaging shows time-dependent internalization of pHrodo Red–labeled IL-23 into macrophages (shown in magenta). Scale bar is 100 µm. **(C)** Quantitation of intracellular fluorescent signal from internalized AF488-labeled mAbs. **(D)** Quantitation of intracellular fluorescent signal from internalized pHrodo Red–labeled IL-23. **(E)** Localization of pHrodo Red–labeled IL-23 (shown in magenta) and AF488-labeled mAb (shown in green) at 20-hour culture time point. Scale bar is 50 µm. **(F)** For lysosome co-localization experiments, macrophages were incubated with SiR-Lysosome instead of CellTracker Deep Red. Localization of pHrodo Red–labeled IL-23 (shown in orange), AF488-labeled guselkumab (shown in green), and SiR-Lysosome (shown in magenta) at 20-hour culture time point. White triangles indicate incidences of guselkumab, IL-23, and lysosome co-localization. Scale bar is 10 µm. All data shown are representative of 2 independent experiments.

Guselkumab and hIgG1 IC were observed to bind to the surface of the macrophages and become internalized in a time-dependent manner (**Figure 4A**). Quantitation of intracellular mAb fluorescence showed similar kinetics for internalization and fluorescence intensity for guselkumab and hIgG1 IC, with detectable intracellular mAb fluorescence starting at approximately 4 hours (**Figure 4C**). The level of intracellular fluorescence intensity for AF488-labeled risankizumab was equivalent to background fluorescence seen in cells cultured in the absence of any mAbs, indicating a lack of binding to the cell surface and therefore no subsequent internalization. Furthermore, we observed time-dependent pHrodo Red fluorescence in the presence of guselkumab, indicating delivery of IL-23 to acidic intracellular compartments (**Figures 4B, D**). Except for rare outliers, internalization of IL-23 was not observed for cells cultured in the presence of IL-23 alone, IL-23 with risankizumab, or IL-23 with hIgG1 IC. Overlay of confocal images demonstrated co-localization of internalized guselkumab and IL-23 (**Figure 4E**).

To further assess subcellular localization of internalized guselkumab and IL-23, CD64^+^ macrophages were incubated with AF488-labeled guselkumab, pHrodo Red–labeled IL-23, and SiR-Lysosome. SiR-Lysosome is a cell-permeable peptide substrate that fluoresces when cleaved by Cathepsin D, a protease present in lysosomes where immune complexes are degraded (51, 52). SiR-Lysosome labeling was strong in many CD64^+^ macrophages, indicating broad expression of Cathepsin D, and fluorescent signal from internalized guselkumab and IL-23 was abundant (**Supplementary Figure S6**; **Figure 4F**). In cells with more moderate SiR-Lysosome uptake, where regions of probe enrichment could be unambiguously resolved, we observed clear incidents of co-localization between guselkumab, IL-23, and the lysosomal marker (**Figure 4F**), consistent with delivery to lysosomal compartments.

### 2.5 Guselkumab demonstrates enhanced functional potency compared to risankizumab in a co-culture of IL-23–producing CD64^+^ myeloid cells and IL-23–responsive cells

**Figure 5:**
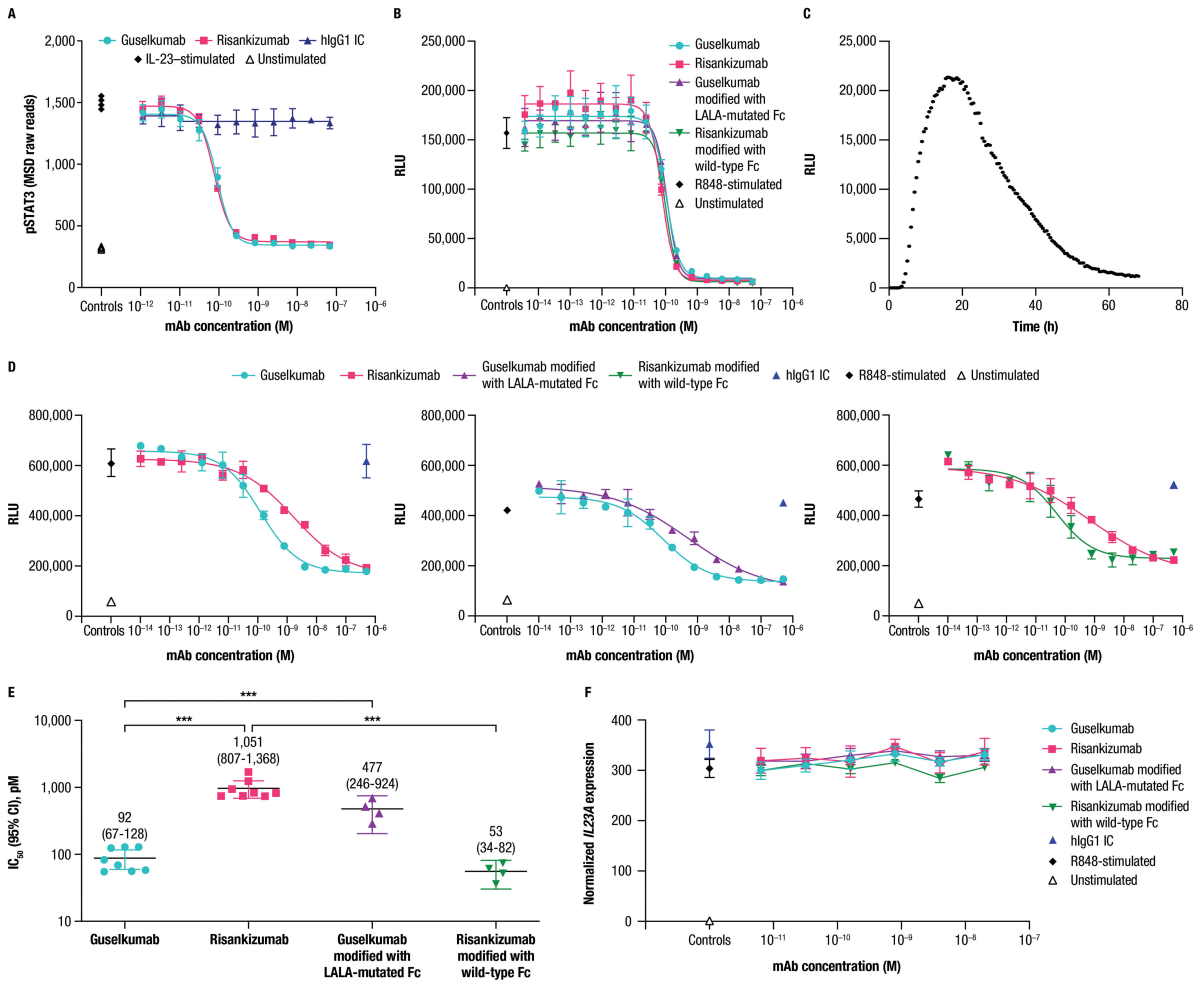
Guselkumab demonstrates enhanced functional potency compared to risankizumab in a co-culture of IL-23–producing CD64^+^ myeloid cells and IL-23–responsive cells. **(A)** Human PBMCs were cultured in the presence of anti-CD3 antibody and IL-1β for 4 days. Guselkumab, risankizumab, or hIgG1 IC were pre-incubated with IL-23 for 1 hour at room temperature, and then added to the PBMCs to give a final IL-23 concentration of 5 ng/mL. Following a 30-minute incubation, cells were lysed and pSTAT3 was measured using the MSD phospho-STAT panel kit. Representative results from 4 independent experiments are shown. **(B–E)** IL-23 signaling was measured in IL-23 reporter cells engineered to express luciferase under a STAT3-inducible promoter. **(B)** Conditioned medium was obtained from THP-1 cells that were stimulated with R848 to promote secretion of IL-23. Guselkumab, risankizumab, hIgG1 IC, guselkumab modified to contain LALA mutations in the Fc domain, or risankizumab modified to contain a wild-type Fc were pre-incubated with the conditioned medium for 1 hour at room temperature, and then added to the IL-23 bioassay cells. Following a 5-hour incubation, evidence of IL-23 signaling was assessed by addition of luciferase substrate and measurement of luminescent signal. Data shown are representative of 2 independent experiments. **(C)** Kinetics of luminescence of IL-23 bioassay cells co-cultured with THP-1 cells following R848 stimulation. Data shown are representative of 3 independent experiments. **(D)** Luminescence of IL-23 bioassay cells co-cultured with THP-1 cells stimulated with R848 in the presence of mAbs. For each experiment, two anti-IL–23p19 subunit mAbs could be evaluated per 96-well plate: guselkumab versus risankizumab, guselkumab versus guselkumab modified with LALA-mutated Fc, and risankizumab versus risankizumab modified with wild-type Fc. Luminescent signal was measured at the 16-hour time point. Plotted data are representative of 4 independent experiments. **(E)** Average IC_50_ values from across 4 independent experiments described in **(D)**. Error bars represent 95% CIs. **(F)** Measurement of *IL23A* transcript by quantitative PCR from co-culture assay system described in **(D)** at 6-hour time point. Plotted data are representative of 3 independent experiments. ***p ≤ 0.001.

We assessed potency of guselkumab and risankizumab for inhibiting IL-23 signaling *in vitro* using human peripheral blood mononuclear cells (PBMCs) cultured in the presence of anti-CD3 antibody and IL-1β for 4 days. Stimulation of these cells with recombinant IL-23 led to phosphorylation of STAT3, a key proximal signaling event downstream of the IL-23 receptor (11). Guselkumab and risankizumab both inhibited IL-23–induced phosphorylation of STAT3 in a dose-dependent manner (**Figure 5A**). A hIgG1 IC antibody with a wild-type Fc domain had no effect on IL-23 signaling. Potency for inhibiting IL-23 signaling was similar for guselkumab and risankizumab, with average IC_50_ values (95% CI) of 80 (69-94) pM and 59 (47-74) pM, respectively. These observations of comparable potency for inhibition of IL-23 signaling are in line with guselkumab and risankizumab binding to IL-23 with similar high affinity (**Supplementary Table S1** and **Supplementary Figure S7**).

Following our observations that guselkumab binding to CD64 conferred unique functions related to IL-23 capture and delivery to endolysosomal compartments, we sought to understand how binding to CD64 could impact potency for inhibition of IL-23 signaling. To this end, we developed an assay to measure biologically active IL-23 produced by CD64^+^ myeloid cells in co-culture with IL-23 reporter cells that express luciferase in response to IL-23 signaling through the IL-23 receptor. THP-1 is a human monocyte cell line with constitutive expression of CD64 that can be upregulated following priming with IFNγ, similar to primary human monocytes (**Supplementary Figure S3C**). Stimulation of IFNγ-primed THP-1 cells with R848 promoted secretion of native IL-23 as measured in THP-1 conditioned medium by signaling in the IL-23 reporter cells (**Figure 5B**).

Guselkumab and risankizumab were shown to have similar potency for inhibition of native IL-23 present in conditioned medium from IFNγ-primed THP-1 cells stimulated with R848 (**Figure 5B**). This is consistent with the observation of similar potency for guselkumab and risankizumab for inhibiting signaling induced by exogenous recombinant IL-23 in primary human PBMCs (**Figure 5A**). Potencies for inhibition of IL-23 present in the conditioned medium were also similar for a modified version of guselkumab containing LALA mutations in the Fc domain and a modified version of risankizumab with a wild-type Fc domain (**Figure 5B**).

We next assessed the potency of guselkumab and risankizumab for inhibiting IL-23 signaling in the co-culture assay. When IFNγ-primed THP-1 cells were stimulated with R848 in co-culture with IL-23 reporter cells, a peak in luminescent signal was observed at approximately 16 hours (**Figure 5C**); therefore, we focused on this time point for evaluation of mAb potency. Strikingly, we observed approximately 10-fold greater potency for guselkumab compared to risankizumab, with average IC_50_ values (95% CI) of 92 (67-128) pM and 1,051 (807-1,368) pM, respectively (**Figures 5D, E**). This difference in functional potency appeared to be mediated through mAb binding to FcγRs (likely CD64), as a modified version of guselkumab with a LALA-mutated Fc domain had decreased potency with an average IC_50_ value (95% CI) of 477 (246-924) pM, and a modified version of risankizumab with a wild-type Fc domain showed an increase in potency with an average IC_50_ value (95% CI) of 53 (34-82) pM (**Figures 5D, E**). In contrast, hIgG1 IC did not inhibit IL-23 signaling. Expression of *IL23A* in the co-culture was further assessed via quantitative PCR, confirming that mAb binding to CD64 did not alter *IL23A* expression by R848-stimulated THP-1 cells (**Figure 5F**).

## 3 Discussion

Human genetic associations and the transformational efficacy of anti–IL-23 therapeutic mAbs implicate the IL-23 pathway as a critical pathogenic driver in PsO, PsA, and IBD (53). Myeloid cells are a key source of IL-23, and CD64 has recently emerged as a surface marker of IL-23–producing myeloid cells residing within inflamed tissues in PsO and IBD (20, 29, 30). Our complementary analyses of bulk transcriptomic datasets from PsO and IBD showed increased expression of CD64 and IL-23 transcripts in inflamed tissue, and our analyses of single-cell transcriptomic datasets further indicate that CD64^+^ myeloid cells are a primary source of IL-23 in inflamed tissue.

In this study, guselkumab and risankizumab, therapeutic mAbs that target the IL-23p19 subunit, were clearly differentiated in their ability to engage FcγRs, with only guselkumab demonstrating Fc-dependent binding to CD64, the sole FcγR capable of high-affinity binding to monomeric IgG1. The ability of guselkumab to bind to CD64 and the observation that CD64 is expressed, both at the transcriptional level and as a protein on the surface of cells that produce IL-23 in inflamed tissue, led us to the hypothesis that a proportion of guselkumab might be positioned within inflamed tissue to bind and neutralize IL-23 at its cellular source of production. *In vitro* assays using exogenous recombinant IL-23 confirmed that guselkumab, when bound to CD64 on the surface of myeloid cells via its Fc domain, retained the ability to bind to IL-23 via its Fab domain. We further observed the ability of guselkumab, but not risankizumab, to bind to CD64 on the surface of IL-23–producing myeloid cells and simultaneously capture endogenous IL-23 secreted locally by these cells. These functional attributes were unique to guselkumab, as risankizumab was unable to bind to CD64.

CD64 has been reported to internalize following binding to monomeric human IgG or when cross-linked, such as by polyvalent antibody-antigen immune complexes or cross-linking antibodies (45–49). Monomeric IgG1 and CD64 complexes have been reported to enter an internalization-recycling pathway (45, 46). However, when CD64 is cross-linked by polyvalent immune complexes or by CD64-targeting polyclonal antibodies, trafficking to lysosomal compartments has been reported (45, 48, 49). In our *in vitro* studies, we observed internalization of hIgG1 IC and guselkumab–IL-23 complexes within CD64^+^ macrophages. Notably, we also observed guselkumab–IL-23 complexes within lysosomal compartments. As binding of guselkumab–IL-23 complexes to CD64 would not be expected to induce CD64 cross-linking, it appears that cross-linking is not a strict requirement for delivery of IL-23 to lysosomes. While our studies do not preclude the possibility that some internalized guselkumab–IL-23 complexes might be recycled back to the cell surface, the detection of these complexes within lysosomal structures supports a hypothesis that guselkumab can mediate both removal and degradation of IL-23 from the extracellular microenvironment in inflamed tissue.

A notable difference between our cellular internalization studies and those from previously published reports is experiment duration. In prior studies, rapid internalization of CD64-specific antibody within 10 minutes was observed in the context of cross-linking (45, 48, 49). In our studies, the kinetics of internalization were evaluated over 24 hours, and internalization was first detectable at approximately 4 hours following monovalent binding of IgG1 IC or guselkumab–IL-23 complexes to CD64. Given these observations, it is possible that the process of internalization following monovalent engagement of CD64 is mediated by a mechanism different than that triggered following CD64 cross-linking by multivalent complexes. FcγRs are identified as activating or inhibitory receptors based on their immunoreceptor tyrosine-based activating motifs or inhibitory motifs, and can mediate effector functions including antibody-dependent cell-mediated cytotoxicity, antibody-dependent cellular phagocytosis, and cytokine release (27, 54). While CD64 is classified as an activating receptor, we did not observe cellular activation, as measured by cytokine secretion, following guselkumab or hIgG1 IC binding to CD64 on primary human monocytes. Induction of cytokine secretion was observed only when CD64 was cross-linked with CD64-specific polyclonal antibodies. Thus, in both internalization and cytokine secretion studies, the outcome of guselkumab binding to CD64 is distinct from that which occurs following CD64 cross-linking. Furthermore, guselkumab or hIgG1 IC binding to CD64 did not enhance the cytokine secretion profile of myeloid cells stimulated with TLR ligands. The lack of cellular activation of myeloid cells following guselkumab binding to CD64 is consistent with the clinical efficacy and favorable safety profile of guselkumab in patients with PsO, PsA, and IBD (23, 55–59).

Considering our observations differentiating guselkumab from risankizumab based on ability to bind CD64, we explored whether binding to CD64 would impact functional potency for inhibiting IL-23 signaling in a co-culture of IL-23–producing CD64^+^ myeloid cells and IL-23–responding cells. Potency for inhibition of recombinant exogenous IL-23–induced signaling in human PBMCs, or native (derived from conditioned medium) exogenous IL-23–induced signaling in IL-23 reporter cells, was comparable for guselkumab and risankizumab and unaffected by modification of the Fc domain. These observations are consistent with prior reports leveraging assay systems that similarly utilized exogenous addition of recombinant IL-23 (60).

However, in a co-culture system of IL-23–producing CD64^+^ myeloid cells with IL-23 reporter cells, guselkumab demonstrated approximately 10-fold higher potency for inhibiting IL-23 signaling than risankizumab. This differential enhancement of potency was dependent on the presence of a wild-type Fc domain, as guselkumab was more potent than a version of guselkumab containing LALA mutations in the Fc domain, and risankizumab was less potent than a version of risankizumab with a wild-type Fc domain. There was no indication in our assay system that CD64 binding altered IL-23 expression or secretion by myeloid cells. Thus, the enhanced potency conferred by wild-type Fc domains and CD64 binding appears to be due to more effective neutralization of locally produced IL-23.

In our cellular internalization studies, hIgG1 IC, which does not bind to IL-23, was observed to internalize within CD64^+^ macrophages. In turn, it is likely that guselkumab can also internalize in the absence of IL-23 binding. This may raise questions of whether guselkumab binding to CD64 may reduce its availability to effectively neutralize IL-23. However, in the co-culture assay, where guselkumab binding to CD64 and internalization could occur, potent inhibition of IL-23 signaling was observed and was indeed more potent than the IL-23 targeting mAbs that lack the capacity to bind CD64. Further, the dosing schedule of guselkumab maintains a maximum serum concentration (C_max_) of 8.09 µg/mL and trough serum concentration (C_trough_) of 1.2 µg/mL (21). The distribution of mAbs into tissues is less than that found in plasma, ranging from 5% to 15.7% in intestine and skin, respectively (61). Thus, the relatively large systemic pool of guselkumab should maintain adequate distribution to inflamed tissue, where CD64 and IL-23 expression are increased. It is challenging to directly extrapolate *in vitro* observations to clinically meaningful differences in patients as there are no direct head-to-head comparisons of guselkumab and risankizumab in a clinical trial setting. While guselkumab and risankizumab have demonstrated efficacy in the treatment of PsO, PsA, UC, and CD (21–24), further studies would be warranted to explore clinical differences between guselkumab and risankizumab (e.g., impact on inhibiting progression of structural joint damage in PsA, impact on axial symptoms in PsA, differences between induction dosing regimens/route of administration to achieve high levels of clinical response in IBD) and how these differences may correlate with CD64 binding capacity and ability to neutralize IL-23 at its cellular source.

Two additional IL-23p19 subunit–targeting mAbs that were not included in this study are tildrakizumab, which is approved for the treatment of PsO (62), and mirikizumab, which is currently approved for the treatment of UC and CD (63). Notably, while tildrakizumab contains an IgG1 wild-type Fc domain and has the ability to bind CD64, it is reported to be much less potent in its ability to bind IL-23 and inhibit IL-23–induced signaling (60). Mirikizumab is an IgG4 antibody with F234A and L235A mutations in the Fc domain that diminish its ability to bind to FcγRs, including CD64 (64, 65). Thus, within the class of IL-23p19 subunit–targeting therapeutic mAbs, guselkumab has unique molecular attributes (the combination of both high-affinity binding to IL-23 and the ability to bind to CD64) that enable highly potent capture and neutralization of IL-23 at its cellular source, which may enhance inhibition of IL-23 within inflamed tissue.

In summary, findings from the *in vitro* assay systems described here differentiate guselkumab and risankizumab based on their capacity to bind CD64. Binding to CD64 enabled unique Fc-dependent functional capabilities of guselkumab, ultimately leading to enhanced potency for inhibiting IL-23 signaling. These *in vitro* data support a hypothesis that, through binding to CD64, a proportion of the available guselkumab may be ideally positioned within the inflamed tissue microenvironment, where CD64^+^ IL-23–producing myeloid cells are increased, to potently neutralize IL-23 at its cellular source of production. In turn, through binding CD64, guselkumab may be optimally situated within the interface between IL-23–producing cells and IL-23–responsive cells, to enhance inhibition of IL-23 signaling in treating immune-mediated diseases.

## 4 Methods

### 4.1 Bulk transcriptomics analyses

PsO RNA-sequencing data were obtained from GSE121212 (37). Read counts from 28 patients with PsO and 38 healthy controls from 5 mm punch biopsies of lesional and non-lesional skin (from patients with PsO) and healthy skin (from controls) were included in the analysis. IBD RNA-sequencing data were obtained from GSE193677 (36). Read counts of rectal biopsies from patients with CD (115 inflamed, 251 non-inflamed), patients with UC (136 inflamed, 164 non-inflamed), and non-IBD controls (225 non-inflamed) were used for analysis. Data were converted to log_2_ counts per million with the edgeR package for plotting and statistical analysis (66).

### 4.2 Single-cell transcriptomic analyses

Single-cell transcriptome data were obtained from previous publications that investigated inflammatory skin diseases (data available at ArrayExpress: E-MTAB-8142) (38) and CD (data available at GEO: GSE134809) (39). The annotations used for cells and tissue types adhered to classifications in the original publications. SCANPY, a large-scale single-cell gene expression data analysis tool, was used for single-cell analyses and visualization (67). For the skin dataset, which included data from 3 patients with PsO, we retained cells meeting the following criteria: total unique molecular identifier counts >1,000; total genes >400, and percentage of mitochondrial reads <20%. For the ileum tissue dataset from patients with CD, 2 patients (Patients 5 and 6) were excluded due to low cell count, thus our analyses were performed on data from 9 patients. Furthermore, within each dataset, raw counts for each gene were normalized to the total counts per cell followed by log transformation to represent gene expression levels.

### 4.3 IL-23 proteins

Recombinant human single-chain IL-23 was purchased from R&D Systems (1290-IL). Recombinant human heterodimeric IL-23 was produced in-house. The coding sequence for the p19 chain was synthesized with a glycine-serine linker followed by a His6 tag at the C-terminus. The coding sequence for the p40 chain was synthesized and each gene was cloned into plasmid pcDNA3.4 (ThermoFisher). HEK293-6E cells were co-transfected with each IL-23 expression construct according to the manufacturer’s instructions (ThermoFisher), and secreted IL-23 protein was purified using a HisTrap excel column (Cytiva) and a HiLoad 26/600 Superdex 200 column (Cytiva). For the assay evaluating IL-23 internalization, recombinant human heterodimeric IL-23 was labeled with the pH-sensitive fluorogenic dye pHrodo Red (Invitrogen) according to the manufacturer’s instructions.

Recombinant human IL-23 heterodimer containing site-specific biotinylation was produced in insect cells. The coding sequence for the mature p19 chain (residues 19-189) was synthesized with the GP67 secretion signal at the N-terminus, and a Gly-Ser linker followed by an Avi tag, TEV protease site, and His8 tag at the C-terminus. The coding sequence for the mature p40 subunit (residues 23-328) was synthesized with the GP67 secretion signal at the N-terminus and each sequence was cloned downstream of a separate promoter in the pFastBacDual vector (ThermoFisher) for production via secreted co-expression in Sf9 insect cells. Secreted IL-23 protein was purified using a HisTrap excel column. The His tag was cleaved with TEV protease and IL-23 was purified using a HisTrap HP column and a HiLoad Superdex 200 column. Purified IL-23 was biotinylated on the C-terminal Avi tag by incubation with Tris buffer plus ATP and biotin (5 mM Tris, 10 mM MgCl_2_, 10 mM ATP, and 0.5 mM biotin at pH 7.5) in the presence of 1:10 (w/w) His-tagged BirA biotin ligase O/N at 4°C, followed by another round of purification using a HisTrap HP column, desalting on a HiPrep 26/10 column, and concentrated and polished using a HiLoad 26/600 Superdex 200 column.

### 4.4 Monoclonal antibodies

Guselkumab was produced as a clinical drug substance at Janssen. Clinical grade risankizumab was purchased from BAP US, Inc. The versions of risankizumab with a wild-type Fc region and guselkumab with a LALA-mutated Fc region were generated at Janssen. Control human wild-type human IgG1 mAb (anti-respiratory syncytial virus) was expressed in HEK293 cells. The mAbs were captured from clarified cell culture supernatants using MabSelect PrismA (Cytiva) and the ÄKTA Pure system (GE Healthcare) and then further purified by preparative SEC using HiLoad 16/600 Superdex 200 pg. For experiments utilizing fluorescently labeled mAbs, mAbs were labeled with AF488 (Thermo Fisher Scientific) according to the manufacturer’s instructions.

### 4.5 Surface plasmon resonance studies

SPR experiments were performed using a Biacore T200 optical biosensor (GE HealthCare). Biosensor surfaces were prepared by amine-coupling goat anti-human IgG Fcγ–fragment specific antibody (Jackson ImmunoResearch Laboratories, Inc., 109-005-098) in 10 mM sodium acetate (pH 4.5) to the surface of a C1 sensor chip (Cytiva). Goat anti-human antibody (~870 RU) was immobilized in each flow cell. All kinetic experiments were run at 37°C using HEPES buffered saline plus surfactant P20 buffer (10 mM HEPES, 150 mM NaCl, 0.05% surfactant P20 at pH 7.4) supplemented with 100 ug/mL BSA. Anti–IL-23 mAbs were captured (30-40 RU) on the anti-human Fcγ surface and analyte injection (recombinant human single-chain or heterodimeric IL-23) followed in a single-cycle kinetic mode (0.4-10.0 nM). The association was monitored for 3 minutes at 50 μL/min and dissociation was monitored for 90 minutes. Regeneration of the sensor surface was achieved with 3 pulses of a 10-second injection of 0.85% H_3_PO_4_ at 100 μL/min. Data were processed using Biacore T200 software. Double reference subtraction (subtracting buffer injection from the reference-subtracted curves for analyte injections) was performed and responses were globally fitted using a 1:1 interaction model. The lower limit of quantitation for the dissociation rate constant (*k_d_*) was estimated using the 5% decay rule as previously described (68, 69).

### 4.6 KinExA studies

*In vitro* binding affinities of guselkumab and risankizumab for recombinant human IL-23 were determined using a KinExA analysis. Serial dilutions of single-chain IL-23 or heterodimeric IL-23 (15 nM-80 fM) were prepared in the presence of a constant concentration of mAb. Assays using heterodimeric IL-23 were performed using 4 different mAb concentrations (0.32, 1.6, 8, and 40 pM). Assays using single-chain IL-23 were performed with 1.5 pM of mAb in triplicate. Titrations of mAb–IL-23 complexes were incubated at room temperature (~22°C). Samples were incubated for 24-72 hours, depending on the antibody concentration, to reach equilibrium. After incubation, the samples were run on a KinExA3200 or 4000 instrument (Sapidyne Instruments) to assess free mAb in the reaction (70). The data were fit with a 1:1 binding model using KinExA Pro software. Global analysis (n-curve analysis) was performed to obtain the affinities and the 95% CIs.

### 4.7 Signal transducer and activator of transcription 3 phosphorylation assay

Cryopreserved PBMCs from healthy donors were resuspended at 2 to 6 × 10^5^ cells/mL in XF-T Cell Expansion Medium supplemented with penicillin/streptomycin, and 100 ng/mL IL-1β (BioLegend) and cultured in tissue culture flasks coated with an anti-CD3 mAb (BD Pharmingen, 555329) for 4 days at 37°C in 5% CO_2_. On Day 4, PBMCs were collected, washed, and incubated in RPMI supplemented with 0.1% BSA for approximately 4 hours at 37°C in 5% CO_2_. Next, 6 × 10^4^ cells in RPMI-BSA (20 µL) were transferred into each well of a 384-well plate. Test mAbs were serially diluted in culture medium, pre-incubated with recombinant human IL-23 heterodimer for 1 hour at room temperature, and added to the PBMCs to give a final IL-23 concentration of 5 ng/mL. The cells were stimulated for 30 minutes at 37°C, then assay plates were transferred onto ice for 5 minutes followed by cell lysis (1% Triton X-100, 150 mM NaCl, 20 mM Tris pH 7.5, 1 mM EGTA, 1 mM EDTA). Levels of phosphorylated STAT3 in cell lysates were measured using the Meso Scale Discovery (MSD) phospho-STAT panel kit (MSD) and a Meso Sector S 600 plate reader. Phosphorylated STAT3 levels (raw MSD plate reads) were plotted versus mAb concentration in GraphPad Prism (version 9). IC_50_ values were averaged from 4 independent experiments utilizing PBMCs from a total of 3 donors.

### 4.8 IL-23 bioassay

IL-23 luciferase reporter bioassay cells (Promega) were cultured in DMEM (Cytiva) with 10% FBS (Corning), 200 µg/mL hygromycin B (Invitrogen), and 600 µg/mL Geneticin (G418; Invitrogen). THP-1 cells (InvivoGen) were cultured with 10% FBS in RPMI-1640 (Sigma), supplemented with L-glutamine (Sigma) and penicillin/streptomycin (Sigma).

For generating conditioned medium containing endogenously produced IL-23, THP-1 cells were pre-treated with 10 ng/mL IFNγ (R&D Systems) overnight then stimulated with 8 µg/mL R848. Culture supernatant (conditioned medium) was collected 18 hours later.

To test potency of mAbs against endogenous IL-23 in conditioned medium, 5 × 10^3^ IL-23 bioassay cells in 20 µL of antibiotic-free DMEM with 10% FBS (assay medium) were added to a sterile 384-well opaque plate (Greiner Bio). Next, 20 µL of mAbs diluted in bioassay culture medium at 4-fold final assay concentration and 20 µL of THP-1–conditioned medium were combined, incubated for 30 minutes at 37°C, and then 20 µL of this mixture was added to cells. Plates were incubated for 5 hours at 37°C with 5% CO_2_ and then equilibrated at room temperature for 10 minutes. Lastly, an equal volume of reconstituted Bio-Glo (Promega) was added to each cell sample and incubated for 5 minutes at room temperature. Plates were read on an Envision 2105 plate reader (PerkinElmer).

For co-culture experiments, THP-1 cells were pre-treated with 10 ng/mL IFNγ overnight. Then, in a sterile 96-well opaque plate (Corning), 2.5 × 10^5^ pre-treated THP-1 cells and 2 × 10^4^ IL-23 bioassay cells were combined per well with 8 µg/mL R848, in the presence or absence of mAbs at the concentrations specified. Plates were incubated for 16 to 17.5 hours at 37°C with 5% CO_2_. Luminescent signal was measured as previously described.

For evaluation of the kinetics of luminescence in co-cultures in the absence of mAbs, the assay was performed as previously described except that beetle luciferin (Promega) was included in the culture as a non-lytic substrate to enable continuous luminescence reading on a BioTek Cytation 5 plate reader (Agilent). Luminescent signals were plotted in GraphPad Prism (version 8). IC_50_ values were averaged from 4 independent experiments.

### 4.9 Quantitative PCR of *IL23A* in IL-23 bioassay co-culture

To assess *IL23A* transcript levels in the co-culture assay, cells were prepared as described previously. At 6 to 6.5 hours post co-culture, cells were washed with PBS, pelleted, and frozen at –80°C. RNA was extracted using RNeasy (Qiagen) and converted to cDNA following the manufacturer’s instructions (Applied Biosystems). Reactions were prepared using Taqman probes for *IL23A* (Thermo Fisher Scientific), *TBP* (Thermo Fisher Scientific), and master mix (Thermo Fisher Scientific) according to the manufacturer’s instructions. Plates were cycled and read using a Quantstudio Flex 12k (Thermo Fisher Scientific). Cycling parameters were 20 seconds at 95°C for 1 cycle, followed by 40 cycles of 1 second at 95°C then 20 seconds at 60°C. Fold-change expression was calculated using the ΔΔCq method and normalized to untreated controls.

### 4.10 Homogeneous time-resolved fluorescence assays

Guselkumab, risankizumab, and hIgG1 IC were assessed for binding to CD64 (FcγRI), CD32a (FcγRIIA), CD32b (FcγRIIB), and CD16 (FcγRIII) using a cellular homogeneous time-resolved fluorescence (HTRF) assay (PerkinElmer, Cisbio) performed according to the manufacturer’s protocol. Briefly, individual FcγRs were expressed in HEK293 and fused with SNAP-tag. Cells were then labeled with SNAP-Lumi-Tb substrate. Pre-labeled cells were thawed, washed, and resuspended in 1.1 mL of Tag-lite buffer. A total of 10 µL of these pre-labeled cells were seeded per well in an opaque 384-well plate (Greiner Bio-One). mAbs were serially diluted in Tag-lite buffer to 4-fold final concentrations. Five µL of each antibody dilution were added to the cells followed by 5 µL of the IgG-d2 conjugate. The assay plate was covered with an adhesive aluminum plate seal and incubated for 2 hours at room temperature. The plate was then unsealed, and fluorescence was read on a Pherastar FS plate reader (BMG Biotech) at 665 nm and 620 nm. Results were reported as the ratio of signal at 665 nm/620 nm, multiplied by 10^4^, and graphed using GraphPad Prism (version 8.4.2).

### 4.11 Flow cytometry

Dose-dependent binding of mAbs to endogenous CD64 was assessed on human monocytes primed with IFNγ. Monocytes were isolated from human PBMCs (IXCells) using a pan-monocyte isolation kit (Miltenyi Biotec). Cells were cultured overnight at 37°C in RPMI (Lonza Bioscience) supplemented with 10% FBS, endotoxin-free penicillin and streptomycin, and recombinant human IFNγ (50 ng/mL; R&D Systems). IFNγ-primed monocytes were subsequently incubated with a titration of AF488-labeled mAbs diluted in stain buffer (BD Biosciences) for 30 minutes on ice. Cells were then washed with stain buffer, and simultaneously labeled with Ghost viability dye (Tonbo) and blocked with FcR-blocking reagent (Miltenyi Biotec) for 15 minutes on ice. Without further washing, surface receptors were detected with anti-human CD14 clone M5E2 (PECy7; Biolegend, 301814), anti-human CD16 clone 3G8 (BV605; Biolegend, 302040), anti-human CD32 clone 3D3 (PE; BD Biosciences, 552884), and anti-human CD64 clone 10.1 (BV510; Biolegend, 305014). For CD64 blocking experiments, IFNγ-primed monocytes were pre-incubated with unlabeled goat polyclonal antibodies specific to CD64 (R&D Systems, AF1257) or isotype control (R&D Systems, AB-108-C) for 30 minutes on ice. AF488-labeled antibodies were added directly into a solution with the monocytes and blocking antibody and incubated for an additional 30 minutes on ice.

To evaluate capture of recombinant biotinylated IL-23, IFNγ-primed monocytes were incubated with mAbs at a concentration of 0.1 µg/mL for 30 minutes on ice. Cells were then washed and incubated with a titration of biotinylated IL-23 for 30 minutes on ice. Cells were washed again, and captured IL-23 was detected with streptavidin (SAV-BV421, BD Biosciences). Cells were also stained with anti-human CD14 clone m5E2 (PE/Cy7; Biolegend, 301814) and Ghost viability dye (Tonbo). Cells from experiments utilizing IFNγ-primed monocytes were acquired using BD FACS Canto.

To evaluate capture of endogenous, locally produced IL-23, CD14^+^ primary human monocytes were cultured in the presence of GM-CSF (2.5 ng/mL; R&D Systems) and IFNγ (50 ng/mL; R&D Systems) for 6 days to induce differentiation into inflammatory monocytes (71). Inflammatory monocytes were subsequently incubated with AF488-labeled mAbs (0.3 µg/mL) and stimulated with the TLR ligands LPS (100 ng/mL; InvivoGen) and R848 (5 µg/mL; InvivoGen) to promote production of endogenous IL-23. After 20 hours of incubation, cells were washed, and captured IL-23 was detected with a biotinylated anti–IL-23p40 mAb (Thermo, clone C8.6, 13-7129-85) followed by staining with streptavidin (SAVe450; ThermoFisher). Surface receptors were detected with anti-human CD64 clone S18012C (PE; Biolegend, 399504), and anti-human CD14 clone m5E2 (PE/Cy7; Biolegend, 301814). The samples were acquired on a Cytek Aurora spectral cytometer. Flow cytometry data were analyzed using FlowJo (version 10.7.1 or 10.8.1) and graphed using GraphPad Prism (version 8.4.2).

### 4.12 Human 41-plex cytokine bead assays

A Milliplex 41-plex human cytokine bead assay (Millipore) was used to evaluate secreted cytokines in culture supernatants from CD14^+^ primary human monocytes cultured under different conditions. IFNγ-primed CD14^+^ primary human monocytes were cultured for 24 hours in the presence of guselkumab, risankizumab, a hIgG1 IC, goat polyclonal antibody specific to CD64 (R&D Systems, AF1257), or goat polyclonal control antibody. Secreted cytokines from inflammatory monocytes were also evaluated; inflammatory monocytes were derived from CD14^+^ primary human monocytes cultured in the presence of GM-CSF and IFNγ for 6 days, followed by incubation with guselkumab, risankizumab, or hIgG1 IC and stimulated with LPS and R848. The Milliplex 41-analyte kit was assessed with a MAGPIX multiplex reader using xPONENT software (version 4.3.309.1). Analyte concentrations were determined using Belysa immunoassay curve fitting software (MilliporeSigma, version 1.1.0). Each analyte was evaluated at the dilution that placed the data nearest to the middle of the standard curve, and values below the limit of detection were set to the limit of detection for log_2_ fold-change calculations. Data were graphed using GraphPad Prism (version 8.4.2. or 9).

### 4.13 Live-cell confocal microscopy

Live-cell confocal microscopy was used to assess whether binding of guselkumab to CD64 leads to internalization of IL-23. Macrophages were grown from PBMC-derived monocytes (Hemacare/Charles River Laboratories Cell Solutions, Inc) in medium containing 50 ng/mL GM-CSF (Peprotech) in T150 flasks for 6 days at 37°C and 5% CO_2_, with a fresh GM-CSF medium change at Day 4. Macrophages were lifted and stained with CellTracker Deep Red (Invitrogen) and Hoechst33342 (Invitrogen) to label cytoplasm and nuclear regions, respectively. Stained cells were washed, seeded into 384-well plates in medium containing 50 ng/mL GM-CSF and 100 ng/mL IFNγ (Peprotech), and incubated at 37°C and 5% CO_2_ for 24 hours. CD64 expression was confirmed by imaging and flow cytometry with anti-human CD64 clone S18012C (PE; Biolegend, 399503) or the IC clone MOPC-21 (PE; Biolegend, 400111). Separately, after 24 hours, macrophages were treated with 10 nM pHrodo Red–labeled IL-23 alone or with 10 nM AF488-labeled guselkumab, risankizumab, or hIgG1 IC, or PBS. Images were acquired over 24 hours on the Opera Phenix High-Content Screening System (PerkinElmer) at 37°C and 5% CO_2_. Fluorescence from AF488 and pHrodo Red was measured in cytoplasmic regions. The readout from image analysis was single-cell mean fluorescence intensity, which was averaged across a given well and time point. Before averaging, outlying single cells were identified and removed using a modified z-score >3.5 (72). Data were graphed using GraphPad Prism (version 8.4.2).

To assess subcellular localization of internalized IL-23 and guselkumab, macrophages were treated for 20 to 22 hours with 10 nM pHrodo Red–labeled IL-23 and 10 nM of AF488-labeled guselkumab, risankizumab, or hIgG1 IC, or PBS. Cells were stained with 50 nM SiR-Lysosome reagent (Cytoskeleton Inc.) for 2 hours before imaging, and single time point images were acquired on the Opera Phenix High-Content Screening System (PerkinElmer) at 37°C and 5% CO_2_. Comparably stained cells with distinct SiR-Lysosome–labeled compartments were examined for co-occurrence of pHrodo Red–labeled IL-23 and AF488-labeled mAbs.

### 4.14 Statistics

Expression of *FCGR1A*, *IL23A*, and *IL12B* were compared in lesional versus non-lesional samples (PsO) and inflamed versus non-inflamed samples (CD and UC) with a 2-sample t-test using the rstatix package (v0.7.0).

For mAb potency in the human PBMCs and co-culture assay, dose-response curve parameters were calculated for each treatment group within the experiment using 4-parameter logistic regression. Log_10_ IC_50_ estimates were averaged by treatment group using meta-analysis, which generated mean log_10_ IC_50_ values weighted by the inverse of their standard error, along with 95% CIs. These estimates were then exponentiated back to the original scale for reporting.

For mAb potency in the co-culture assay, random-effects meta-regression was performed on log_10_ IC_50_ values of all treatment groups to test for statistical differences between different treatments; the following pre-specified comparisons were made between treatment groups: guselkumab versus risankizumab, guselkumab versus guselkumab modified with LALA-mutated Fc domain, and risankizumab versus risankizumab with wide-type Fc domain. *P* values were adjusted for multiple comparisons using the False Discovery Rate. All analyses were performed in R v4.3.0. Dose response estimates were generated via the drda package and meta-analysis was performed via the metafor package.

## Data Availability

The datasets presented in this study can be found in online repositories. The names of the repository/repositories and accession number(s) can be found below: https://www.ncbi.nlm.nih.gov/ for GSE121212, GSE193677, and GSE134809; and https://www.ebi.ac.uk/biostudies/arrayexpress for E-MTAB-8142.
