# Supplementary material for: Guselkumab binding to CD64+ IL-23–producing myeloid cells enhances potency for neutralizing IL-23 signaling
Source: Front Immunol. 2025 Mar 12;16:1532852. doi: 10.3389/fimmu.2025.1532852 (PMC11937023; doi:10.3389/fimmu.2025.1532852)
Supplement: Supplementary file 1 [file DataSheet1.docx]

Supplementary Material

Guselkumab binding to CD64^+^ IL-23–producing myeloid cells enhances potency for neutralizing IL-23 signaling

Kacey L. Sachen^1*^, Deepa Hammaker^1^, Indra Sarabia^1^, Brian Stoveken^2^, John Hartman^2^, Kristin L. Leppard^2^, Nicholas A. Manieri^2^, Phuc Bao^1^, Carrie Greving^1^, Eilyn R. Lacy^2^, Matthew DuPrie^2^, Joshua Wertheimer^2^, Janise D. Deming^1^, Joseph Brown^1^, Amy Hart^2^, He (Hurley) Li^2^, Tom C. Freeman^2^, Brice Keyes^1^, Kristen Kohler^1^, Ian White^2^, Nathan Karpowich^2^, Ruth Steele^2^, M. Merle Elloso^3^, Steven Fakharzadeh^3^, Kavitha Goyal^3^, Frédéric Lavie^4^, Maria T. Abreu^5^, Matthieu Allez^6^, Raja Atreya^7^, Robert Bissonnette^8^, Kilian Eyerich^9,10^, James G. Krueger^11^, Dennis McGonagle^12^, Iain B. McInnes^13^, Christopher Ritchlin^14^, Anne M. Fourie^1^

^1^Janssen Research & Development, LLC, San Diego, CA, United States

^2^Janssen Research & Development, LLC, Spring House, PA, United States

^3^Janssen Scientific Affairs, LLC, Immunology, Horsham, PA, United States

^4^The Janssen Pharmaceutical Companies of Johnson & Johnson, Paris, France

^5^University of Miami, Leonard M. Miller School of Medicine, Miami, FL, United States

^6^Hôpital Saint-Louis, Université Paris Cité, Paris, France

^7^Department of Medicine 1, Erlangen University Hospital, Friedrich-Alexander-Universität Erlangen-Nürnberg, Erlangen, Germany

^8^Innovaderm Research Inc, Montréal, Québec, Canada

^9^Medical Center, University of Freiburg, Freiburg, Germany

^10^Karolinska Institute, Department of Medicine – Division of Dermatology and Venereology, Stockholm, Sweden

^11^The Rockefeller University, Laboratory for Investigative Dermatology, New York, NY, United States

^12^University of Leeds, Leeds Biomedical Research Centre, Leeds, UK

^13^University of Glasgow, College of Medical, Veterinary, and Life Sciences, Glasgow, UK

^14^University of Rochester, Center for Musculoskeletal Research, Allergy, Immunology, and Rheumatology Division, Rochester, NY, United States

*** Correspondence:**Kacey L. Sachen

[ksachen@its.jnj.com](mailto:ksachen@its.jnj.com)

# Supplementary Figures

**Supplementary Figure 1.** CD64 and IL-23 transcripts are increased in inflamed tissue of PsO and IBD.


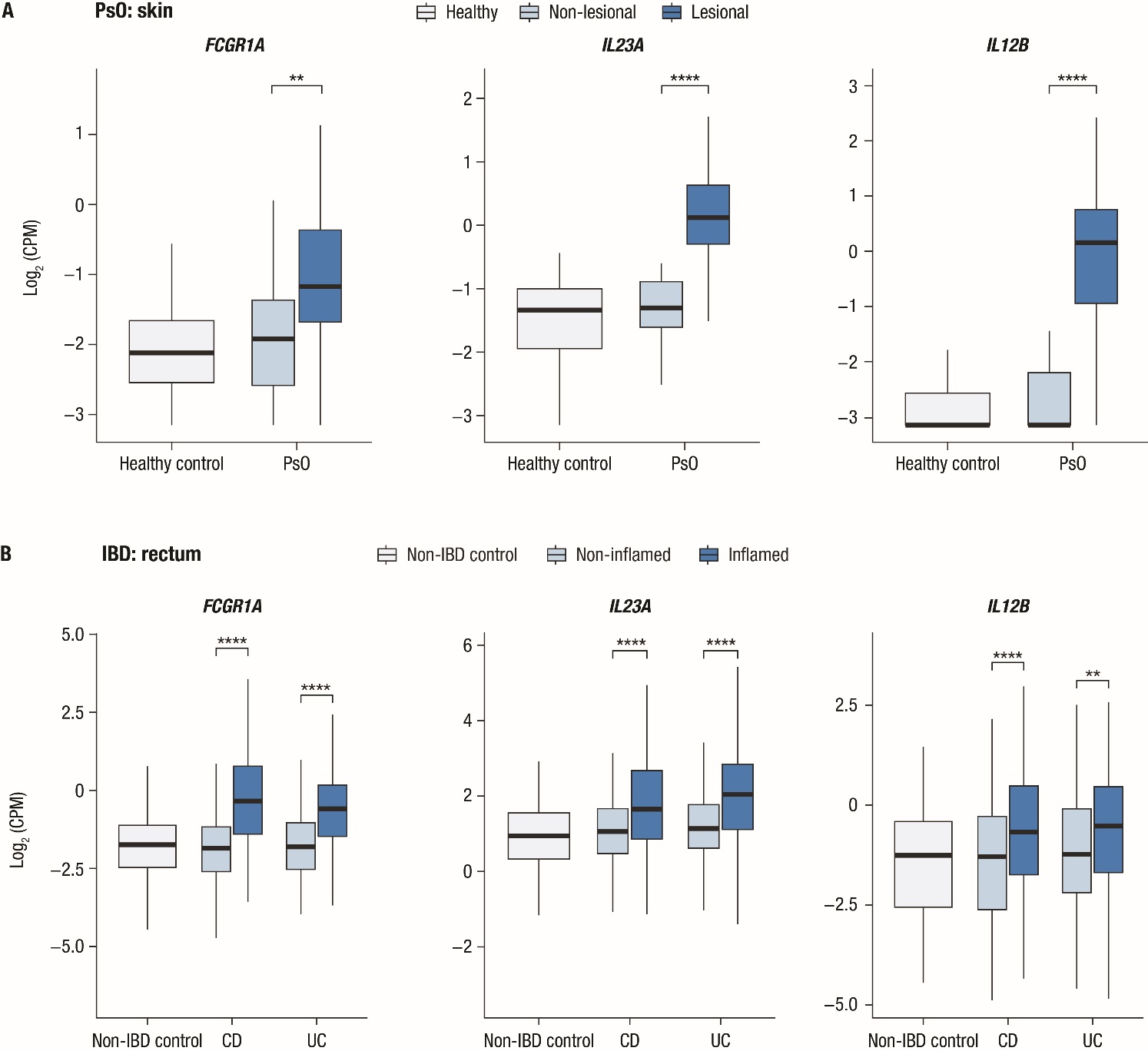


Assessment of *FCGR1A*, *IL23A*, and *IL12B* expression from bulk transcriptomic datasets of (A) skin biopsies from patients with PsO (n = 28 lesional and non-lesional) and healthy controls (n=38) and (B) rectal biopsies from CD (n = 115 inflamed, 251 non-inflamed), UC (n = 136 inflamed, 164 non-inflamed), and non-IBD (n = 225 non-inflamed) patients. All comparisons were evaluated with a 2-sample t-test (**p* < 0.05, ***p* < 0.01, ****p* < 0.001, *****p* < 0.0001).

**Supplementary Figure 2.** Evaluation of guselkumab binding to specific recombinant FcγRs.


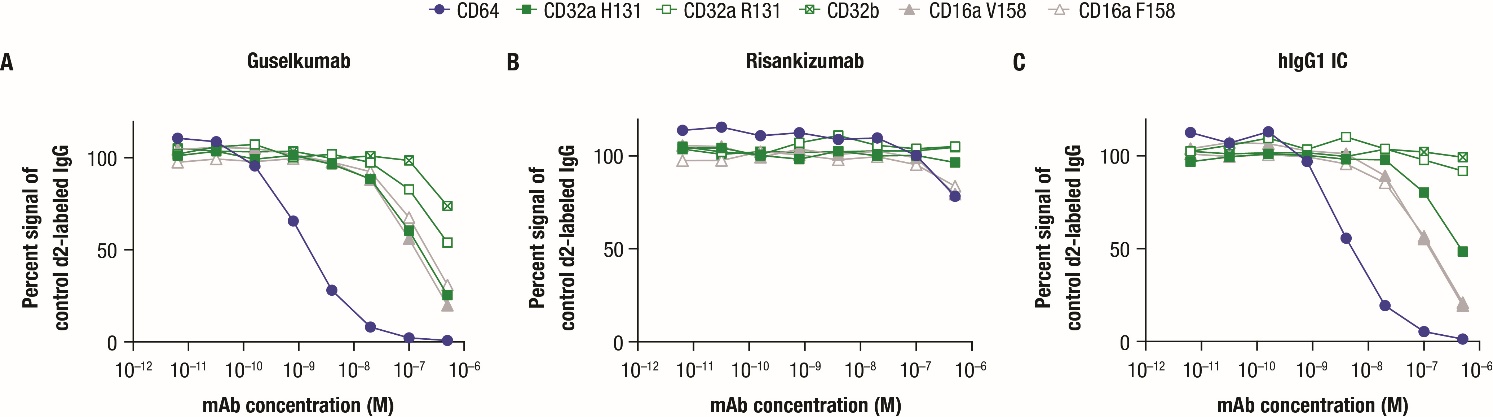


mAb binding to FcγRs was assessed using a cellular HTRF assay. Individual FcγRs were expressed in HEK293 and fused with SNAP-tag. Cells were then labeled with SNAP-Lumi-Tb substrate. Binding of IgG labeled with the d2 acceptor generates a specific FRET, which can be displaced by an unlabeled test antibody that binds to the FcγR. Displacement of control IgG-d2 control was evaluated in the presence of a dose titration of guselkumab (A), risankizumab (B), or hIgG IC (C). Representative data from ≥3 independent experiments are plotted as percent signal of IgG-d2 control.

**Supplementary Figure 3.** CD64 expression by myeloid cells across assay systems.


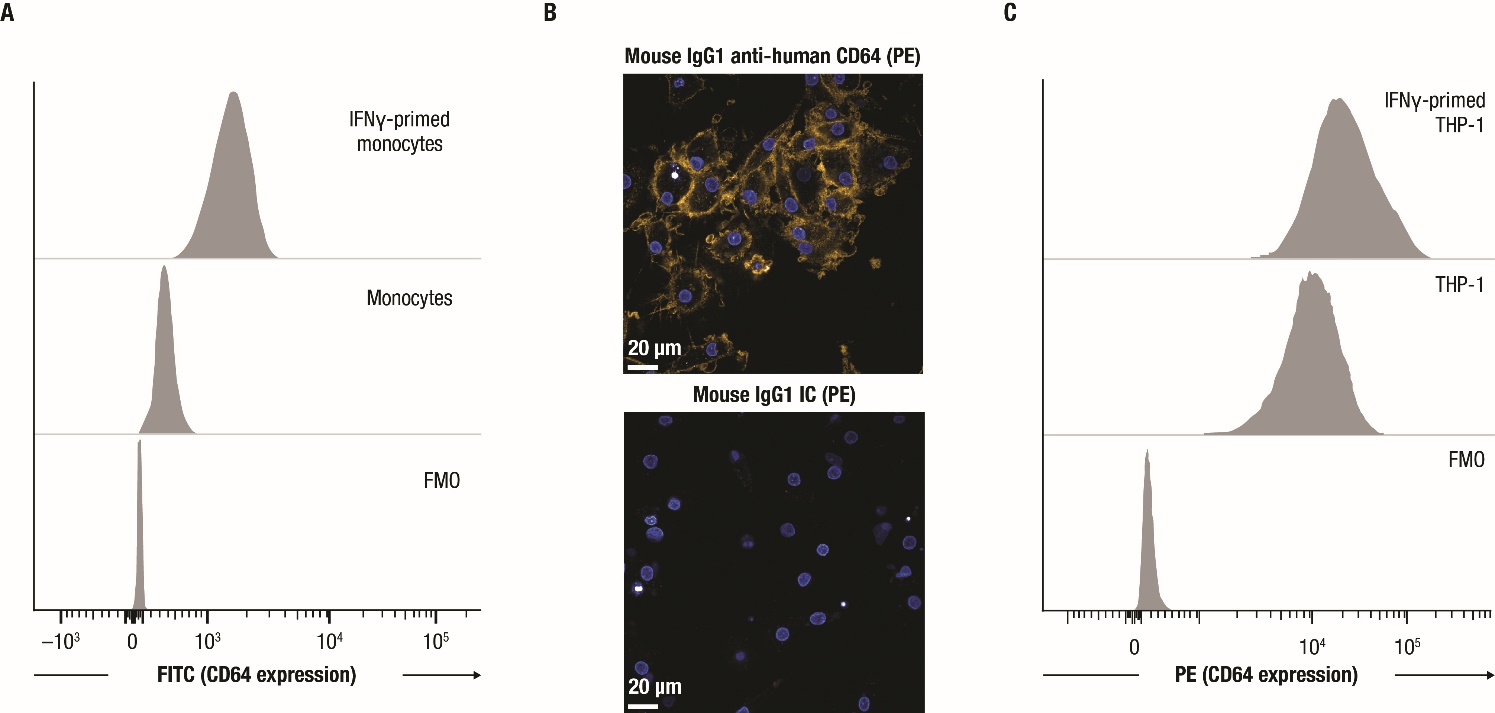


(A) Primary human monocytes were cultured overnight in the presence or absence of IFNγ followed by detection of surface CD64 expression by flow cytometry. Data shown are representative of 3 independent experiments. (B) Primary human monocytes were differentiated into CD64-expressing macrophages by culturing in the presence of GM-CSF for 6 days and were then primed overnight with IFNγ. Live cell fluorescence imaging of surface CD64 expression was performed with high-throughput spinning disk confocal microscopy. Scale bar is 20 µm. Data shown are representative of 2 independent experiments (C) Detection of surface CD64 expression on THP-1 cells by flow cytometry. Data shown are representative of 3 independent experiments.

**Supplementary Figure 4.** Guselkumab binding to primary human monocytes correlates most strongly with level of CD64 expression.


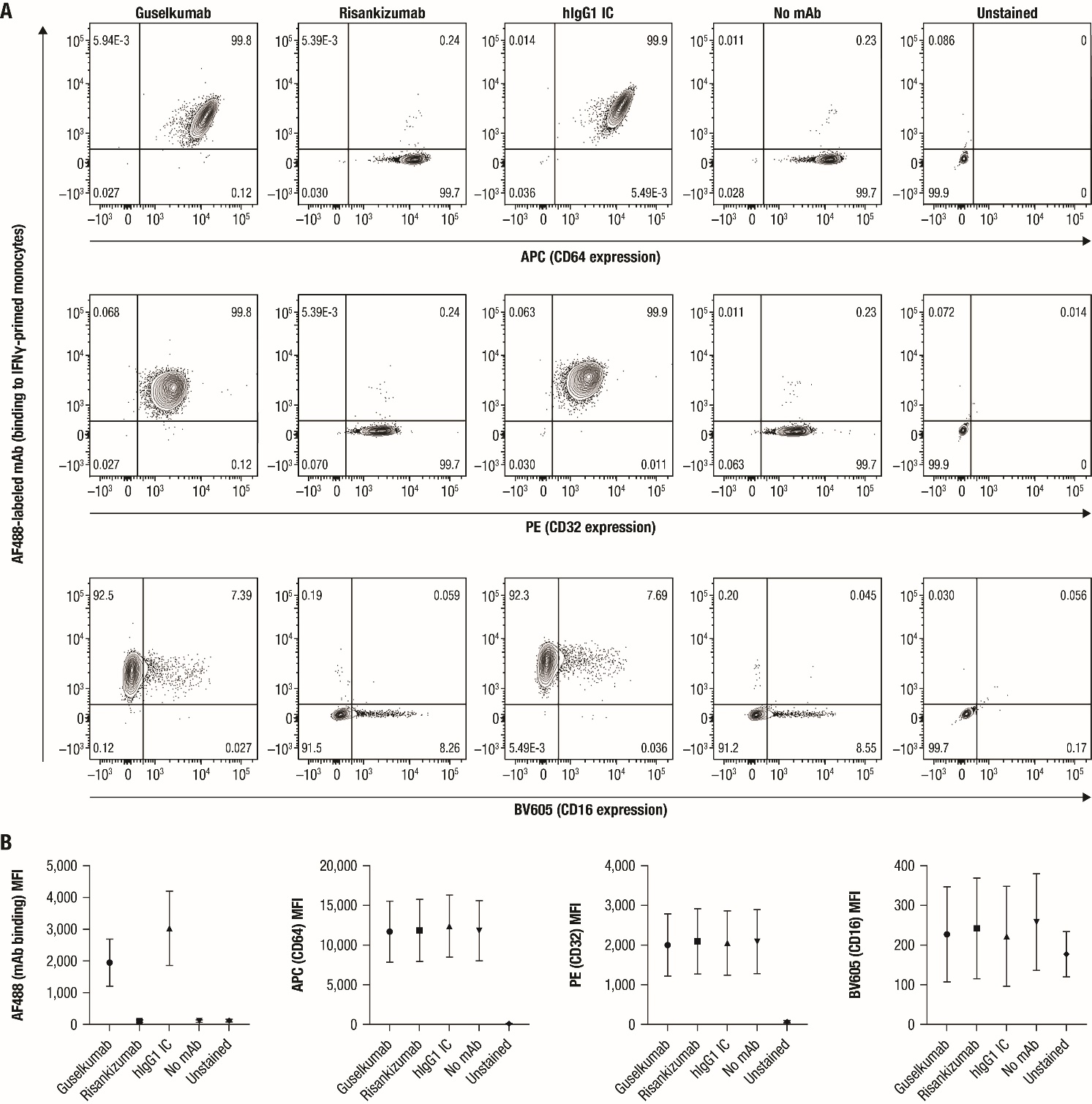


(A) Flow cytometry plots showing correlation of AF488-labeled antibody binding with expression levels of specific FcγRs on IFNγ-primed monocytes. (B) Graphs of flow cytometry data showing geometric mean of AF488-labeled mAb binding, CD64, CD32, and CD16. Error bars indicate the robust standard deviation. Data shown are representative of 3 independent experiments.

**Supplementary Figure 5. Expression of CD64, mAb binding, and IL-23 capture on inflammatory monocytes**

**
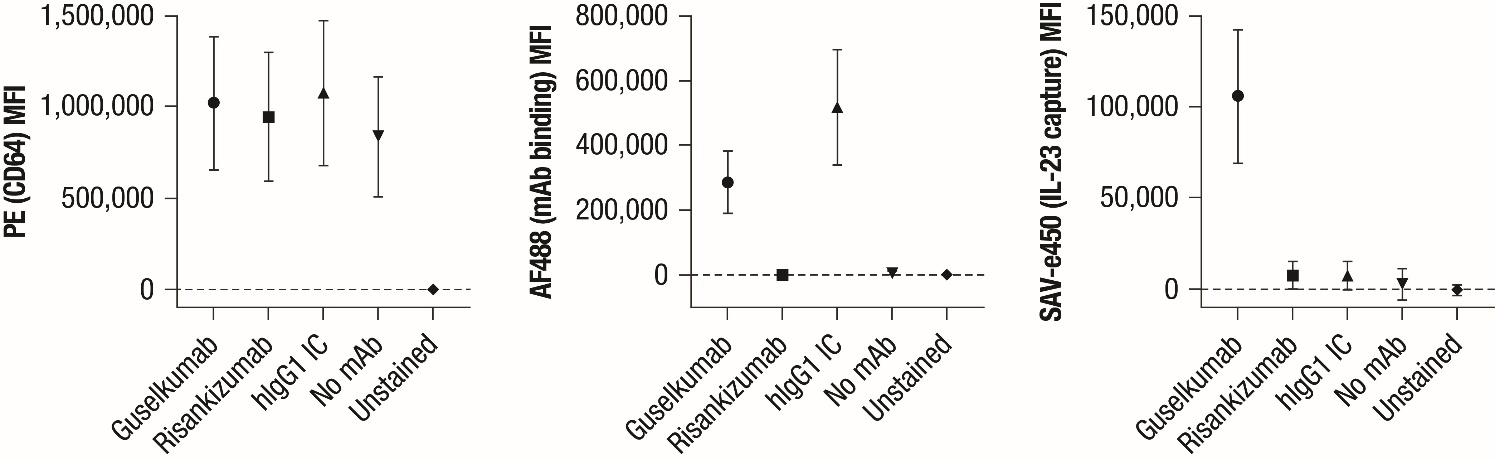
**

Graphs of flow cytometry data presented in Figure 2D showing geometric mean of CD64, AF488-labeled mAb binding, and IL-23 capture. Error bars indicate the robust standard deviation. Data shown are representative of 3 independent experiments.

**Supplementary Figure 6.** Immunofluorescence imaging of human macrophages cultured in the presence of fluorescently labeled guselkumab and IL-23, and labeled with SiR-Lysosome probe and Hoechst stain.


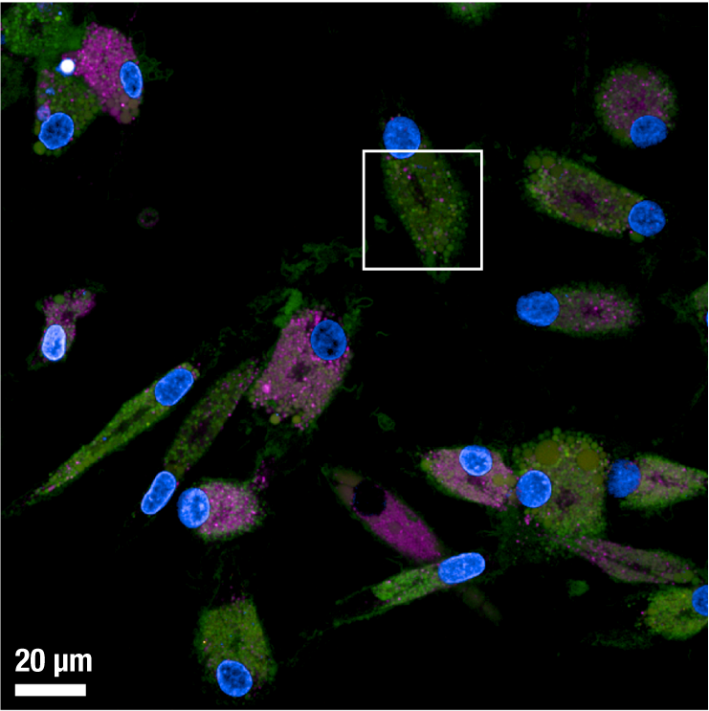


Primary human monocytes differentiated into macrophages as described in Figure 4. Immunofluorescence imaging of pHrodo Red–labeled IL-23 (shown in orange), AF488-labeled guselkumab (shown in green), Hoechst nuclear stain (shown in blue), and SiR-Lysosome (shown in magenta) at the 20-hour culture time point. Scale bar is 20 µm. Data shown are representative of 2 independent experiments.

**Supplementary Figure 7.** Guselkumab and risankizumab bind to IL-23 with similar affinity and kinetics.


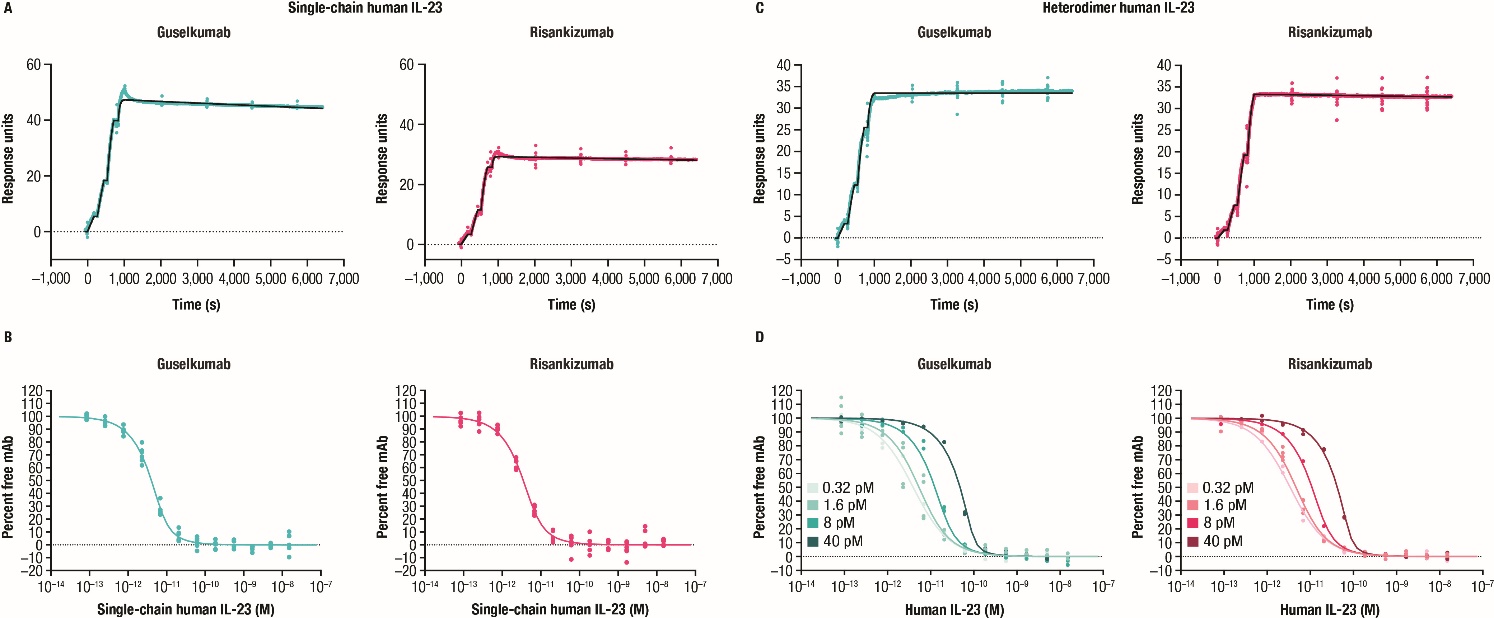


(A and C) SPR sensogram data for antibody binding to single-chain or heterodimer human IL-23. Goat anti-human IgG Fcγ fragment specific antibody was immobilized on the chip. Anti–IL-23p19 antibodies were then captured on the anti-human Fcγ surface followed by IL-23 analyte injection in a single cycle kinetic mode. Sensograms are representative of 4 independent experiments. (B and D) KinExA data for antibody binding to single-chain or heterodimer human IL-23. Serial dilutions of IL-23 were prepared in the presence of a constant concentration of mAb. Titrations of mAb–IL-23 complexes were incubated at room temperature (~22 °C) to reach equilibrium. After incubation, the samples were run on a KinExA instrument to assess free mAb in the reaction. (B) Data shown are the results of one study in which the complex was prepared independently 3 times with 1 mAb concentration which was run in duplicates for a total of 6 titrations. (D) Data shown are the results of one study in which the complex was prepared independently with 4 different mAb concentrations and tested in duplicates.

# Supplementary Table

**Supplementary Table 1.** Binding Affinity and Kinetics of Guselkumab and Risankizumab to Single-chain Human IL-23 and Human IL-23 Heterodimer Using SPR and KinExA

|  | Single chain human IL-23 | | | | Heterodimer human IL-23 | |
| --- | --- | --- | --- | --- | --- | --- |
|  | SPR | | | KinExA | SPR^a^ | KinExA |
|  | ***k_a_* × 10^6^** (95% CI), 1/Ms | ***k_d_* × 10^–5^** (95% CI), 1/s | **K_D_** (95% CI), pM | **K_D_** (95% CI), pM | ***k_a_* × 10^6^** (95% CI), 1/Ms | **K_D_** (95% CI), pM |
| Guselkumab | 2.8 (2.5-3.1) | 1.4 (1.3-1.4) | 4.7 (4.1-5.3) | 1.0 (0.04-2.4) | 3.6 (2.1-5.2) | 3.6 (1.8-6.4) |
| Risankizumab | 4.1 (1.9-6.4) | 1.0 (0.9-1.2) | 2.7 (1.3-4.1) | 1.2 (0.2-2.9) | 2.7 (2.5-2.8) | 3.1 (2.4-4.0) |

^a^*k_d_* of guselkumab and risankizumab to heterodimer IL-23 by SPR assay were beyond limits of quantitation under experimental conditions used. SPR data shown are the average of at least 4 independent experiments. KinExA data shown for single chain IL-23 are the results of one study in which the complex was prepared independently 3 times with 1 mAb concentration which was run in duplicates for a total of 6 titrations. The K_D_ and 95 % CI shown are the result of global fitting of the 3 titration curves shown in Supplemental Figure 7B. KinExA data shown for heterodimer IL-23 are the results of one study in which the complex was prepared independently with 4 different mAb concentrations and tested in duplicates. The K_D_ and 95 % CI shown are the result of global fitting of the 4 titration curves shown in Supplemental Figure 7D.
